# Supplementary material for: Synthesis of bioactive (1→6)-β-glucose branched poly-amido-saccharides that stimulate and induce M1 polarization in macrophages
Source: Nat Commun. 2022 Aug 9;13:4661. doi: 10.1038/s41467-022-32346-5 (PMC9363418; doi:10.1038/s41467-022-32346-5)
Supplement: Supplementary file 1 — Supplementary Information [file 41467_2022_32346_MOESM1_ESM.pdf]

# Supplementary Information

## Synthesis of Bioactive (1→6)-β-Glucose Branched Poly-amido-saccharides that Stimulate and Induce M1 Polarization in Macrophages

Ruiqing Xiao,[1,2,3†] Jialiu Zeng,[4,5†] Eric M. Bressler,[4] Wei Lu,[6] and Mark W. Grinstaff [1,4,*]

[1] Department of Chemistry, Boston University, Boston, Massachusetts 02215, United States

[2] David H. Koch Institute for Integrative Cancer Research, Massachusetts Institute of Technology, Cambridge, Massachusetts 02139, United States

[3] Shenzhen Middle School, Shenzhen, Guangdong 518001, China

[4] Department of Biomedical Engineering, Boston University, Boston, Massachusetts 02215, United States

[5] Lee Kong Chian School of Medicine, Nanyang Technological University, Singapore 308232, Singapore

[6] Tosoh Bioscience LLC, King of Prussia, Pennsylvania 19406, United States

[†] These authors contribute equally

* Email: mgrin@bu.edu

Supplementary Table of Contents

Small Molecule Synthetic Procedures 2

Supplementary Figure 1. Gen-lactam monomer synthesis 2

Supplementary Figure 2. $^1$H NMR spectrum of the polymerization mixture with a [M]/[I] ratio of 100 3

Supplementary Figure 3. $^1$H NMR spectrum of polymer P2 4

Supplementary Figure 4. FTIR spectra of polymers P1-P4 4

Supplementary Figure 5. $^{13}$C NMR spectrum of polymer P2 5

Supplementary Table 1. Characterization of molecular weight of P2' and 3' with triple detectors 5

Supplementary Figure 6. Synthesis of Gen-PASs with *tert*-butylacetyl terminal group 6

Supplementary Table 2. GPC and NMR characterization of Gen-PASs with *tert*-butylacetyl terminal group 6

Supplementary Figure 7. GPC traces of polymer P5-P9 7

Supplementary Figure 8. Calculation of FB values from $^1$H NMR 7

Supplementary Figure 9. Confirmation of endotoxin-free material by chromogenic LAL assay 8

Supplementary Table 3. Characterization of Branched PAS polymers with a FB Value of 30% 9

Supplementary Figure 10. GPC traces of polymers P10'-P13' and P10-P13 9

Supplementary Figure 11. Effect of PMB on the NF-κB/AP-1 activation RAW-Blue cells 9

Supplementary Figure 12. Percentage of CD68+ cells from viable cells in culture 10

Supplementary Figure 13. Representative flow cytometry plots of CD68+ primary macrophages 11

NMR spectra of 2,3,4,2',3',4',6'-hepta-*O*-acetyl-D-gentiobiosyl bromide (**2**) 12

NMR spectra of 3,4,2',3',4',6'-hexo-*O*-benzyl-D-gentiobial (**3**) 13

NMR spectra of Gen-lactam 13

FTIR spectra of Gen-lactam 15

NMR spectra of polymers P1'-P4' 15

NMR spectra of polymers P1-P4 17
NMR spectra of polymers P5-P13 25
NMR spectra of polymers P14'-P16' 30
NMR spectra of polymers P14-P16 31
Supplementary References 32

**Small Molecule Synthetic Procedures**

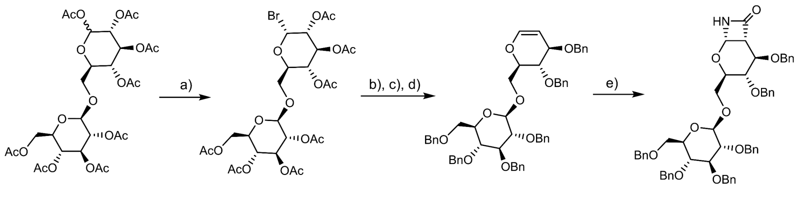


1 2 3 Gen-lactam

**Supplementary Figure 1**. Gen-lactam monomer synthesis. Reagents and conditions: (a) HBr, AcOH, DCM, 0 °C, 55%; (b) Zn, NMI, EtOAc, reflux, 86%; (c) $K_2CO_3$, MeOH, 100%; (d) NaH, BnBr, DMF, 0−25 °C, 90%; (e) TCAI, $CHCl_3$, $BnNH_2$, 39%.

**2,3,4,2',3',4',6'-Hepta-*O*-acetyl-α-D-gentiobiosyl bromide (2).** The procedure was adapted from the literature with minor changes.[1] To a solution of gentiobiose octacetate (**1**) (50g, 73.7 mmol) and acetic anhydride (20 mL) in anhydrous chloroform ($CHCl_3$) (200 mL) was added 33% solution of hydrogen bromide (HBr) in acetic acid (AcOH) (125 mL) slowly at 0 °C. The solution was allowed to stir for 4 h in ice bath, then the reaction mixture was poured into ice-water (500 mL), extracted with $CHCl_3$ (3 x 150 mL). The combined organic layers were washed with a saturated sodium bicarbonate solution (sat. $NaHCO_3$) until neutral pH, and then with brine (200 mL). The organic layer was then dried over sodium sulfate ($Na_2SO_4$), filtered, and concentrated under reduced pressure. The residue was purified by silica gel column chromatography (cyclohexane–ethyl acetate, 2:1) and recrystallization (cyclohexane-diethyl ether) to give compound **2** (28.3g, 55%) as a white solid, $^1$H NMR (500 MHz, $CDCl_3$): δ 6.60 (d, *J* = 4.0 Hz, 1H), 5.51 (t, *J* = 9.7 Hz, 1H), 5.18 (t, *J* = 9.5 Hz, 1H), 5.05 (m, 2H), 4.98 (dd, *J* = 9.6, 8.0 Hz, 1H), 4.77 (dd, *J* = 10.0, 4.0 Hz, 1H), 4.52 (d, *J* = 8.0 Hz,1 H), 4.23 (m, 2 H), 4.11 (dd, *J* = 12.4, 2.4 Hz, 1H), 3.96 (dd, *J* = 11.5, 2.2 Hz, 1H), 3.67 (ddd, *J* = 10.0, 4.7, 2.4 Hz, 1H), 3.59 (dd, *J* = 11.6, 5.1 Hz, 1H), 2.04 (m, 21H); $^{13}$C NMR (125 MHz, $CDCl_3$): δ 170.6, 170.2, 169.8 (2), 169.7, 169.5 169.3 (2), 100.7, 86.5, 73.1, 72.6, 71.9, 70.8, 70.5, 70.2, 68.2, 67.5, 66.6, 61.8, 21.7 (2), 20.6 (5); HRMS (*m/z*): $[M+Na]^+$ calcd. for $C_{26}H_{35}BrO_{17}Na$, 721.0955; found, 721.0950.

**3,4,2',3',4',6'-Hexa-*O*-acetyl-D-gentiobial.** The procedure was adapted from the literature with minor changes.[2] 2,3,4,2',3',4',6'-hepta-*O*-acetyl-α-D-gentiobiosyl bromide (**3**) (24.8 g, 35.4 mmol) was dissolved in ethyl acetate (EtOAc, 350 mL), then activated Zn powder (13.9 g, 212.6 mmol) and *N*-methylimidazole (NMI, 3.0 mL, 37.7 mmol) were added. The mixture was refluxed for 4 hours. After cooling to room temperature, the mixture was filtered through celite and the filtrate washed with sat. $NH_4Cl$ (200 mL), sat. $NaHCO_3$ (200 mL), and brine (200 mL), then dried with $Na_2SO_4$. After filtration, the solvent was evaporated under vacuum. The crude product was purified by flash chromatography (EtOAc-hexane, 1:2) to afford pure 3,4,2',3',4',6'-hexa-*O*-acetyl-D-gentiobial (17.2 g, 86%) as a white solid. $^1$H NMR (500 MHz, $CDCl_3$): δ 6.44 (dd, *J* = 6.2, 1.3 Hz, 1H), 5.28 (m, 1H), 5.19 (t, *J* = 9.4 Hz, 1H), 5.09 (m, 2H), 4.98 (dd, *J* = 9.8, 8.0 Hz, 1H), 4.80 (dd, *J* = 6.2, 3.4 Hz, 1H), 4.56 (d, *J* = 8.0 Hz, 1H), 4.25 (dd, *J* = 12.4, 4.6 Hz, 1 H), 4.20 (td, *J* = 7.0, 3.4 Hz, 1H), 4.12 (dd, *J* = 12.4, 2.4 Hz, 1H), 3.98 (dd, *J* = 11.4, 3.4 Hz, 1H), 3.72 (dd, *J* = 11.4, 6.8 Hz, 1H), 3.68 (ddd, *J* = 9.9, 5.4, 2.8 Hz, 1H), 2.03 (m, 18H); $^{13}$C NMR (125 MHz, $CDCl_3$): δ 170.6, 170.4, 170.2, 169.5, 169.4 (2), 145.6, 100.9, 98.7, 74.8, 72.6, 71.8, 71.0, 68.2, 67.4, 67.2, 67.0, 61.8, 21.0, 20.8, 20.7, 20.6 (3); HRMS (*m/z*): $[M+Na]^+$ calcd. for $C_{24}H_{32}O_{15}Na$, 583.1639; found, 583.1667.

**3,4,2',3',4',6'-Hexa-*O*-benzyl-D-gentiobial (3)**. To a solution of 3,4,2',3',4',6'-hexo-*O*-actyl-D-gentiobial (16.0 g, 28.5 mmol) in methanol (200 mL), $K_2CO_3$ (600 mg, 3.61 mmol) was added. The mixture was stirred at room temperature for 16 hours. The solvent was then evaporated and D-gentiobial was used in the next step without further purification.

To the solution of D-gentiobial (8.8 g, 28.5 mmol) and tetrabutylammonium bromide (462 mg, 1.43 mmol) in DMF (300 mL), NaH (5.40 g, 202.5 mmol) was added slowly at 0 °C. The solution was stirred for 30 minutes at room temperature. After cooling down to 0 °C, benzyl bromide (BnBr, 26.4 mL, 222.6 mmol) was added dropwise and the reaction mixture was warmed up to room temperature and stirred for 48 hours. After quenching the reaction with water, 500 mL EtOAc was added to the solution. The organic phase was washed with water three times and then brine, and dried over $Na_2SO_4$. After filtration, the solvent was evaporated under vacuum. The crude was purified by flash chromatography (EtOAc–hexane from 1: 20 to 1: 6) and yielded compound **3** as white solid (21.9g, 90%). $^1$H NMR (500 MHz, $CDCl_3$): δ 7.40-7.10 (m, 30 H), 6.42 (dd, $J$ = 6.2, 1.2 Hz, 1H), 5.02 (d, $J$ = 10.9 Hz, 1H), 4.94 (d, $J$ = 10.8 Hz, 1H), 4.90 (dd, $J$ = 6.2, 3.0 Hz, 1H), 4.80 (m, 2H), 4.73 (m, 2H), 4.62 (m, 3H), 4.53 (m, 3H), 4.42 (d, $J$ = 7.8 Hz, 1H), 4.22 (m, 2H), 4.14 (m, 1H), 3.88 (dd, $J$ = 11.1, 6.4 Hz, 1H), 3.78 (dd, $J$ = 7.4, 5.4 Hz, 1H), 3.70 (m, 2H), 3.62 (m, 2H), 3.45 (t, $J$ = 8.2 Hz, 1H), 3.42 (ddd, $J$ = 9.4, 4.3, 2.0 Hz, 1H); $^{13}$C NMR (125 MHz, $CDCl_3$): δ 144.6, 138.6, 138.4, 138.2 (2), 138.1, 128.4 (3), 128.3 (2), 128.2, 128.0, 127.9, 127.8, 127.7 (3), 127.6 (2), 104.1, 99.6, 84.7, 82.1, 77.8, 76.2, 75.7, 75.0, 74.9, 74.8, 74.3, 74.2, 73.5, 73.1, 70.3, 68.9, 68.6; HRMS (*m/z*): $[M+Na]^+$ calcd. for $C_{54}H_{56}O_9Na$, 871.3822; found, 871.3837.

**Gen-lactam.** The procedure was adapted from the literature with minor changes.[3] To a solution of **3** (12.4 g, 14.6 mmol) in anhydrous $CHCl_3$ (30 mL), trichloroacetyl isocyanate (TCAI, 3.5 mL, 29.3 mmol) was added at room temperature. The mixture was stirred at room temperature to complete the cycloaddition (monitored by $^1$H NMR). Subsequently, the mixture was diluted with $CHCl_3$ (30 mL) and cooled to -30°C, after which benzylamine ($BnNH_2$, 5.2 mL, 47.4 mmol) in $CHCl_3$ (50 mL) was added slowly, and the mixture was allowed to rise to room temperature. The solvent was then evaporated and the residue was treated with diethyl ether. The crystalline precipitate was removed by filtration and washed with hexane. This filtrate and washings were combined and evaporated under vacuum. The crude was purified by silica gel flash chromatography (EtOAc– cyclohexane from 1: 5 to 1: 2) and recrystallization (cyclohexane-EtOAc) to give Gen-lactam as a white solid (5.1 g, 39%). $^1$H NMR (500 MHz, $CDCl_3$): δ 7.38-7.12 (m, 30 H), 6.10 (s, 1H), 5.46 (d, $J$ = 4.4 Hz, 1H), 4.95 (d, $J$ = 11.2 Hz, 1H), 4.92 (d, $J$ = 10.8 Hz, 1H), 4.79 (m, 3H), 4.66 (m, 3H), 4.51 (m, 4H), 4.42 (d, $J$ = 7.8 Hz, 1H), 4.19 (m, 1H), 4.12 (m, 2H), 3.77 (m, 2H), 3.69 (dd, $J$ = 10.8, 4.6 Hz, 1H), 3.63 (t, $J$ = 9.0 Hz, 1H), 3.58 (t, $J$ = 9.4 Hz, 1H), 3.50 (m, 2H), 3.41 (m, 2H); $^{13}$C NMR (125 MHz, $CDCl_3$): δ 167.0, 138.6, 138.5, 138.0, 137.8 (2), 137.3, 128.5, 128.4 (3), 128.1, 127.9 (4), 127.8, 127.7 (2), 127.6, 104.6, 84.6, 82.3, 77.6, 77.2, 76.7, 76.5, 76.4, 75.7, 75.0, 74.7, 74.5, 73.2, 71.0, 70.4, 68.6, 68.5, 54.6; HRMS (*m/z*): $[M+Na]^+$ calcd. for $C_{55}H_{57}O_{10}N$, 914.3880; found, 914.3916.

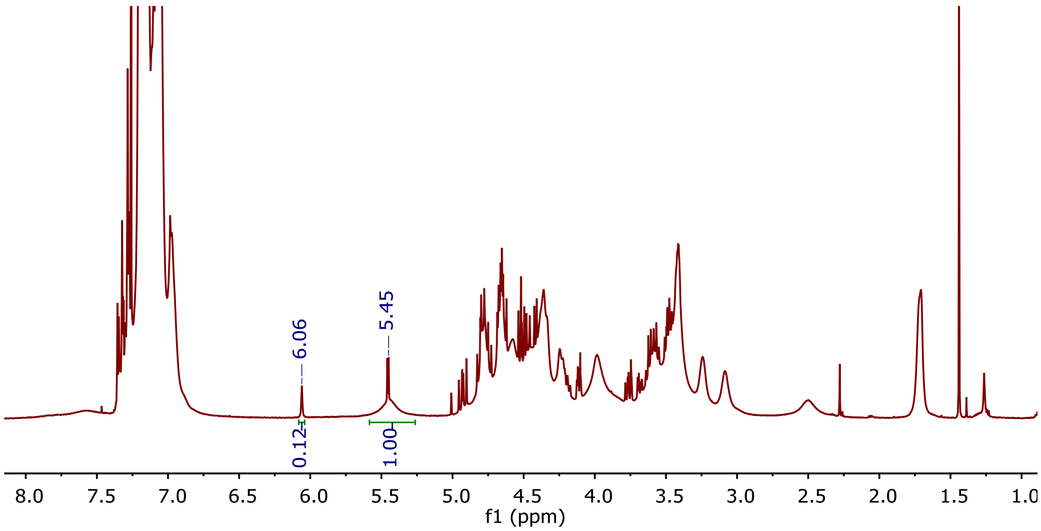


**Supplementary Figure 2** $^1$H NMR spectrum of the polymerization mixture with a [M]/[I] ratio of 100 ($CDCl_3$, monomer conversion was calculated by integration of the proton peaks at 6.06 ppm and 5.45 ppm).

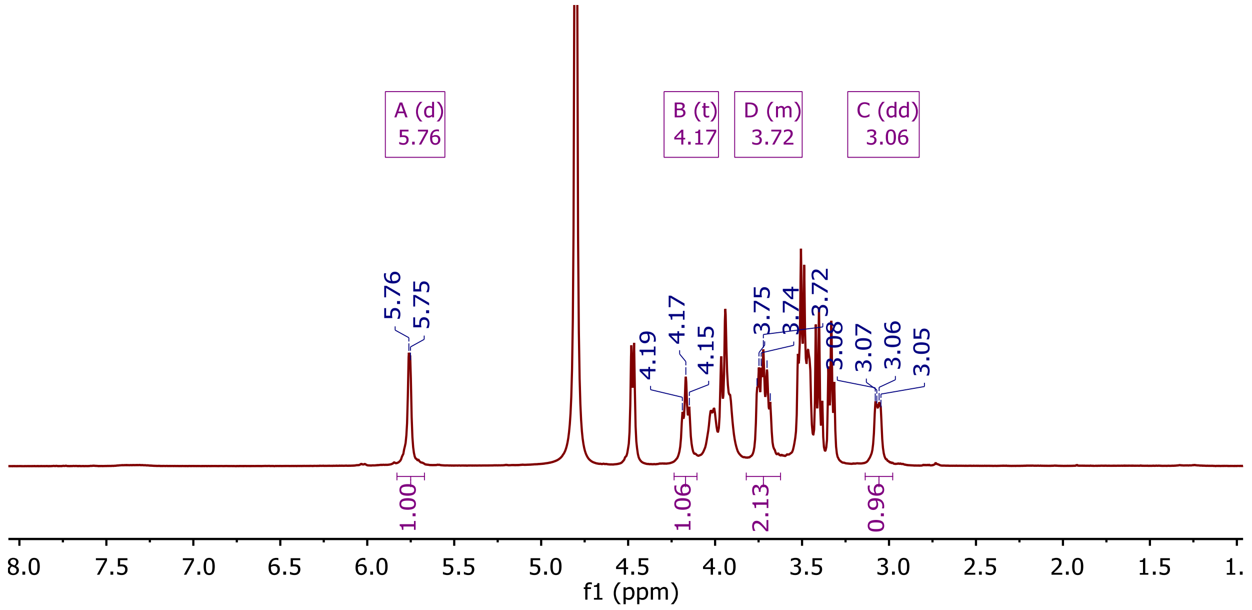


**Supplementary Figure 3** $^{1}$H NMR spectrum of polymer P2 ($D_2O$).

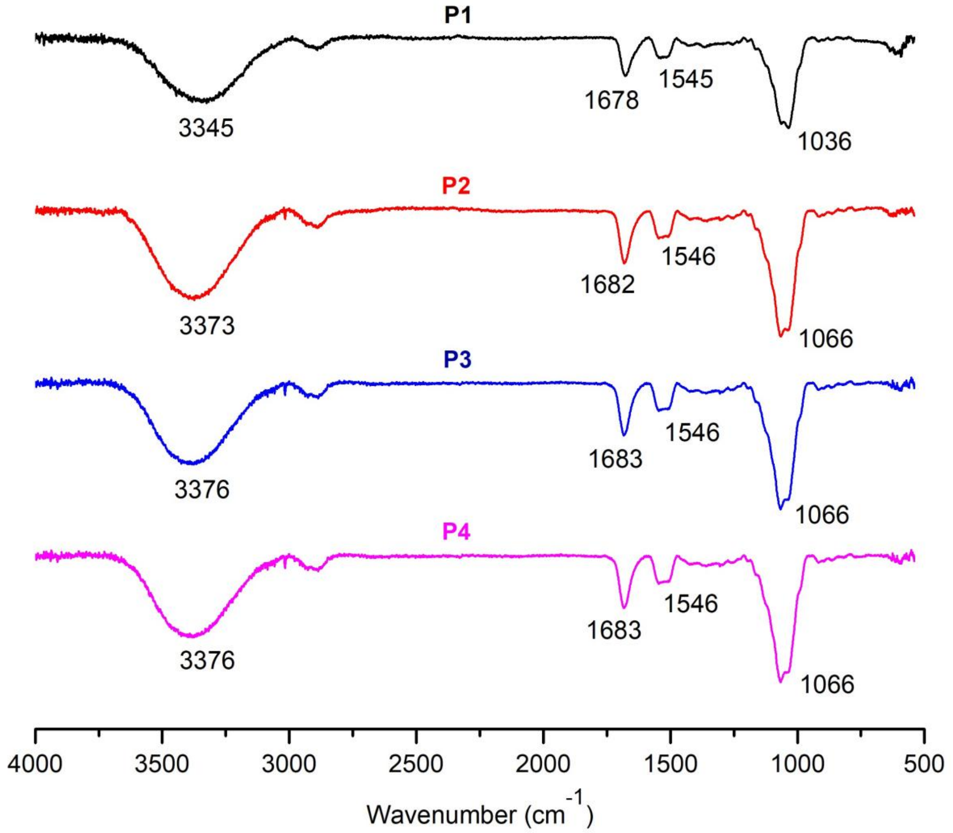


**Supplementary Figure 4.** FTIR of polymers P1-P4.

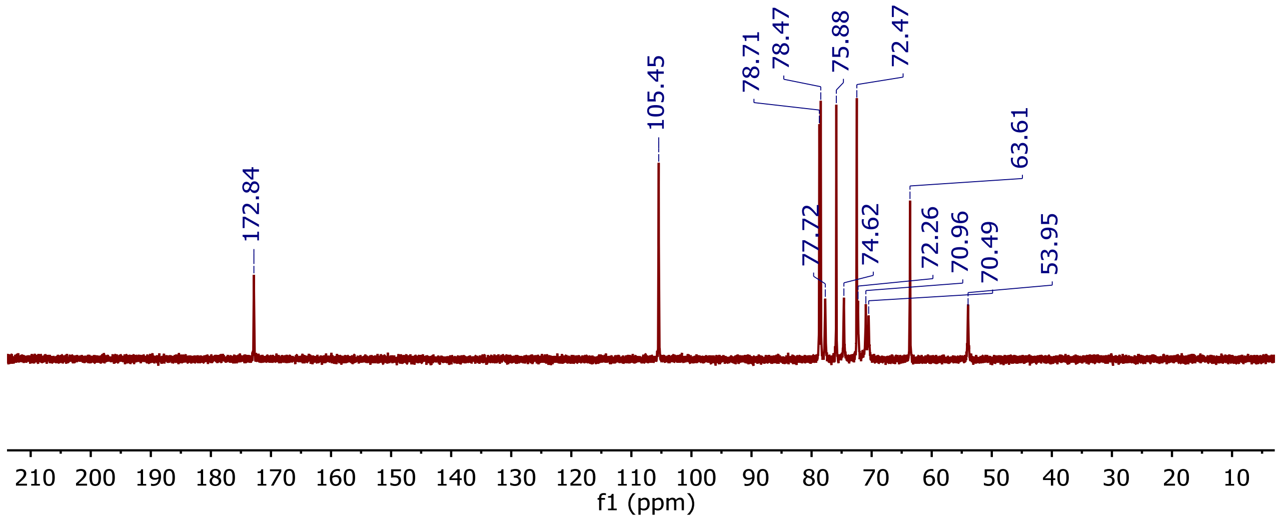


**Supplementary Figure 5** $^{13}$C NMR spectrum of polymer P2 ($D_2O$).

**Discussion of polymer molecular weight**

The $DP_{(GPC)}$ values of P1-P4 are slightly higher than the corresponding $DP_{(GPC)}$ values of the benzylated polymers P1'-P4' (Table 1). This discrepancy in polymer chain lengths may arise from underestimation of the molecular weights of P1'-P4', since branched polymers, in general, possess more compact structure and smaller hydrodynamic volumes compared to the linear polymer standards (polystyrene).[4] In order to confirm this, we measured the molecular weights of P2' and P3' with triple-detection GPC combining refractive index detector (RI), light scattering detector (LS) , and viscometer (VISC) (Tosoh EcoSEC Elite Model HLC-8420).

**Supplementary Table 1. Characterization of molecular weight of P2' and P3' with triple detectors (LS, RI and Viscometer)**

| | Calibration Method | Retention Time [d] | $M_n$ [e] | $M_w$ [e] | $M_z$ [e] | Đ [f] | $R_{g,z}$ [g] | η [h] | $R_h$ [i] | $R_g/R_h$ |
|---|---|---|---|---|---|---|---|---|---|---|
| | LS [a] | | 46,649 | 47,466 | 48,185 | 1.02 | 5.6 | | | |
| P2' | CC [b] | 6.668 | 16,348 | 17,718 | 18,691 | 1.08 | | | | 1.647 |
| | UC [c] | | 50,039 | 50,853 | 51,643 | 1.02 | | 0.052 | 3.4 | |
| | LS | | 90,396 | 94,736 | 97,332 | 1.05 | 7.8 | | | |
| P4' | CC | 6.227 | 29,139 | 34,502 | 37,769 | 1.18 | | | | 1.472 |
| | UC | | 91,184 | 94,965 | 98,659 | 1.04 | | 0.099 | 5.3 | |

[a] light scattering; [b] conventional calibration with RI detector (polystyrene standard); [c] universal calibration with RI detector (polystyrene standard); [d] min; [e] g/mol; [f] $M_w/M_n$; [g] radios of gyration, nm; [h] intrinsic viscosity (IV), dL/g; [i] hydrodynamic radius, nm.

As shown in the Supplementary Table 2, the MW values obtained with conventional calibration (RI, polystyrene standard) are significantly lower than the MW values obtained with light scattering detector, indicating the MW values of benzylated polymers, as measured in Table 1, were underestimated.

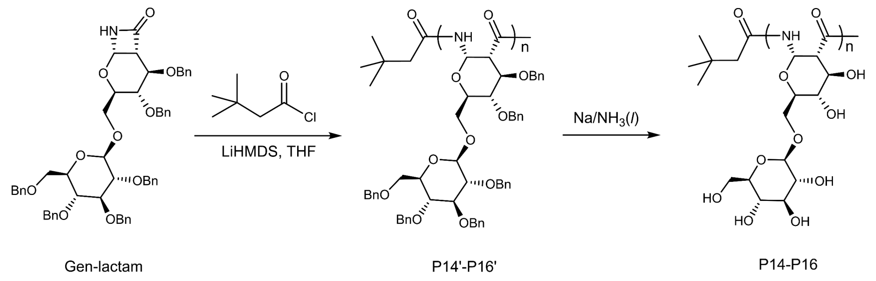


**Supplementary Figure 6.** Synthesis of Gen-PASs with *tert*-butylacetyl terminal group. Reagents and conditions: (a) *tert*-butylacetyl chloride, LiHMDS, THF, 0 °C, yield: 84%-87%; (b) Na, $NH_3$ (*l*), −60 °C, yield: 79%-90%.

In order to further investigate the discrepancy between $DP_{(GPC)}$ of benzylated polymers and deprotected PASs, we performed the polymerization of Gen-lactam using *tert*-butylacetyl chloride as initiator with [M]/[I] ratios of 15, 25, and 50, and obtained benzylated polymers P14'-P16' and deprotected PASs P14-P16 (Supplementary Figure 6). *tert*-Butylacetyl chloride was used as initiator because it has nine methyl protons and enables reliable $DP_{(NMR)}$ analysis by terminal group integration. As we can see from Supplementary Table 2, the $DP_{(GPC)}$ values of P14'-P16' were significantly lower than the [M]/[I] feed ratios, but the $DP_{(NMR)}$ values of P14'-P16' were in good agreement with the [M]/[I] feed ratios, indicating underestimation of MW for benzylated polymers as measured by GPC with conventional calibration with RI detector (polystyrene standard). Again, the $DP_{(GPC)}$ values of deprotected polymers P14-P16 were higher than the $DP_{(GPC)}$ values of P14'-P16', which was consistent with the results for P1'-P4' and P1-P4. However, the $DP_{(NMR)}$ values of P14-P16 were highly consistent with $DP_{(NMR)}$ values of P14'-P16', indicating that degradation of the polymers did not occur during deprotection reactions. Therefore, the discrepancy between $DP_{(GPC)}$ values before and after deprotection is due to the different GPC characterization methods.

**Supplementary Table 2. GPC and NMR characterization of Gen-PASs with *tert*-butylacetyl terminal group**

| Entry | [M]/[I] | $M_{n(theo)}$[a] | $M_{n(GPC)}$[b] | $DP_{(GPC)}$[b] | Đ [c] | $DP_{(NMR)}$[d] | Yield [e] |
|---|---|---|---|---|---|---|---|
| P14' | 15 | 13479 | 9700 | 10.8 | 1.06 | 17.2 | 84 |
| P15' | 25 | 22399 | 16800 | 18.7 | 1.05 | 28.1 | 88 |
| P16' | 50 | 44699 | 35240 | 39.4 | 1.05 | 54.9 | 87 |
| P14 | - | 5364 | 6100 | 17.1 | 1.12 | 18.0 | 79 |
| P15 | - | 8874 | 9600 | 27.1 | 1.14 | 29.7 | 86 |
| P16 | - | 17649 | 17600 | 49.9 | 1.23 | 53.5 | 90 |

[a] Calculated based on [M]/[I] ratios, g/mol. [b] Determined by THF GPC against polystyrene standards for P14'-P16', or aqueous GPC against dextran standards for P14-P16, g/mol. [c] $M_w/M_n$. [d] Measured by terminal group analysis, °. [e] Isolated yield, %.

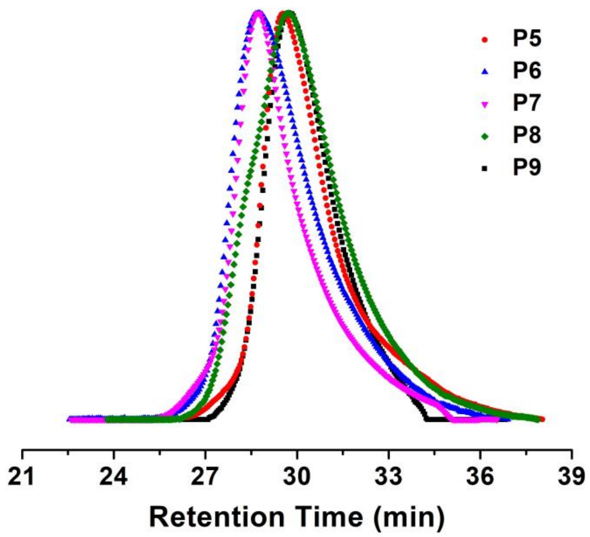


**Supplementary Figure 7**. GPC traces of polymer P5-P9

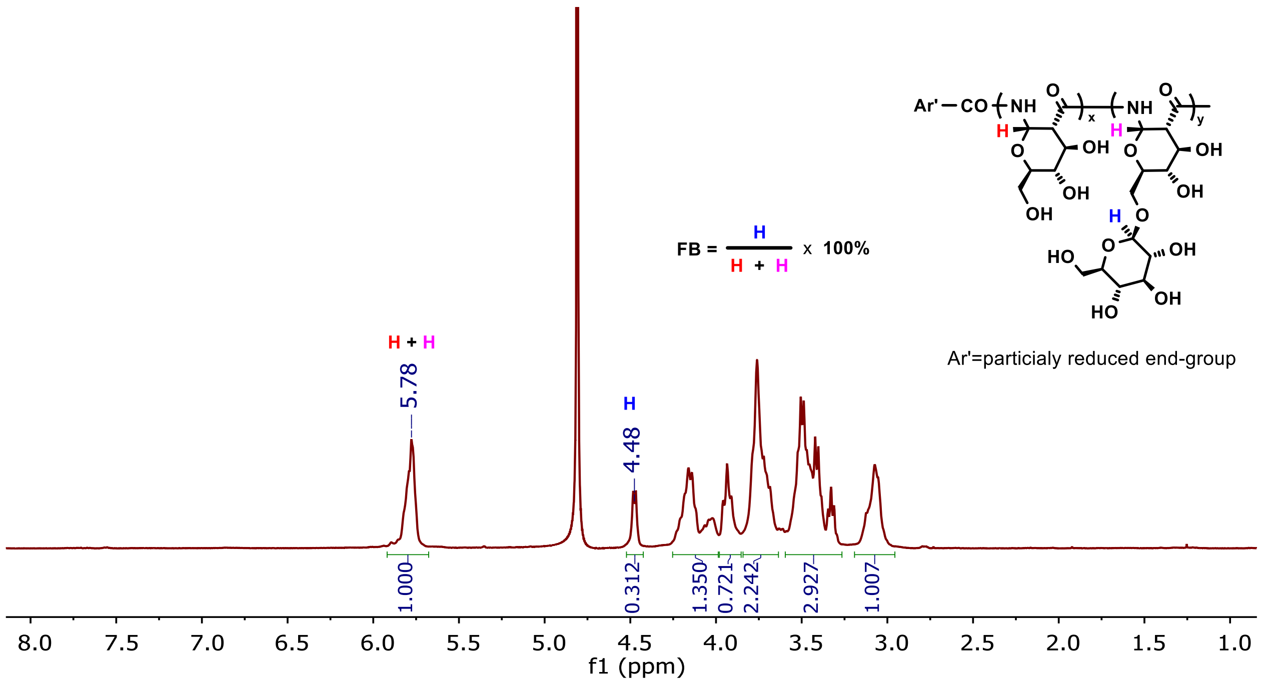


**Supplementary Figure 8.** Calculation of the FB values by integration of the H1' proton of the gentiobiose repeating units (4.48ppm) compared to the H1 protons of both the gentiobiose and glucose repeating units (5.78ppm) along the polymer backbone (P7) ($D_2O$).

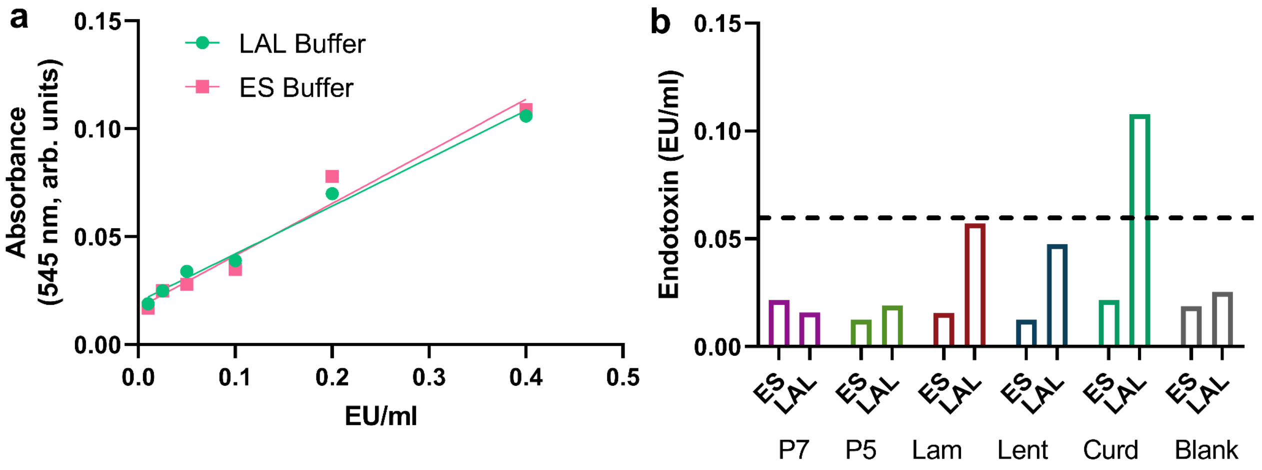


**Supplementary Figure 9:** Confirmation of endotoxin-free material by chromogenic LAL assay. (a) Standard curve of endotoxin from 0.01 EU/ml to 0.4 EU/ml in ES buffer or standard LAL reagent water, (b) endotoxin level of each sample in ES buffer or standard LAL reagent water.

**Supplementary Table 3 Characterization of Branched PAS polymers with a FB Value of 30%**

| Entry | [M]:[I] [a] | $M_{n(GPC)}$ [b] | Đ [c] | FB [d] | $[\alpha]_D^{25}$ [d] | Yield [e] |
|---|---|---|---|---|---|---|
| P10' | 17 | 9700 | 1.08 | N.D. | +71.4 | 95 |
| P11' | 30 | 24800 | 1.05 | N.D. | +72.8 | 87 |
| P12' | 100 | 55900 | 1.06 | N.D. | +72.5 | 91 |
| P13' | 200 | 71600 | 1.05 | N.D. | +73.7 | 85 |
| P10 | - | 3600 | 1.18 | 31.8 | +113.1 | 78 |
| P11 | - | 12100 | 1.41 | 30.6 | +115.2 | 88 |
| P12 | - | 27500 | 1.36 | 29.7 | +113.9 | 92 |
| P13 | - | 46100 | 1.27 | 31.0 | +112.5 | 94 |

[a] Monomer-to-initiator ratios. [b] Determined by THF GPC with polystyrene standards for P10'-P13', or aqueous GPC with dextran standards for P10-P13, g/mol. [c] $M_w/M_n$. [d] Determined by $^1$H NMR analysis. N.D., not determined. [e] Isolated yield, %.

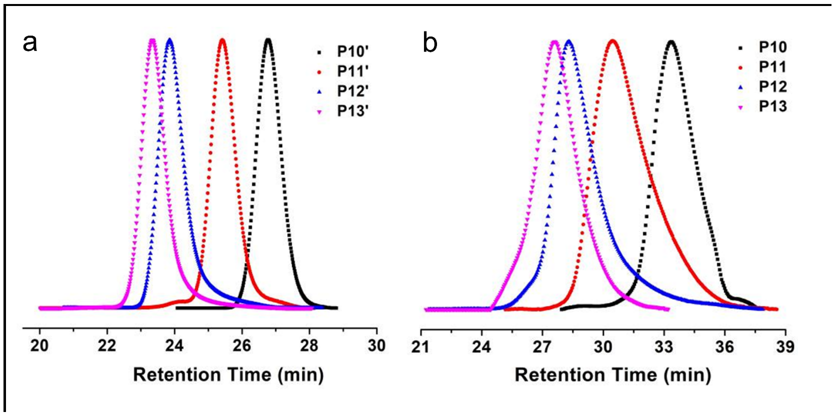


**Supplementary Figure 10**. GPC traces of polymers P10'-P13' and P10-P13. (s) THF GPC traces of polymers P10'-P13'; (b) GPC traces of polymers P10-P13

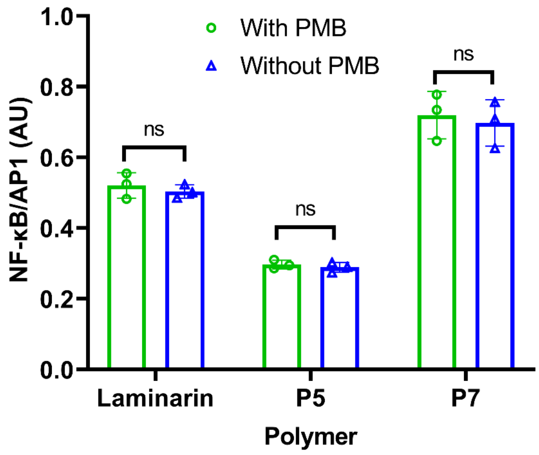


**Supplementary Figure 11.** Effect of PMB on the NF-κB/AP-1 activation RAW-Blue cells induced by Glc-PAS (P5), 30% branched PAS (P7), and Laminarin (Lam). Data are means ± SD of three independent experiments. Data are means ± SD of three independent experiments. ns $p>0.05$, *$p \leq 0.05$, **$p \leq 0.01$ compared to control using two-tailed unpaired t test.

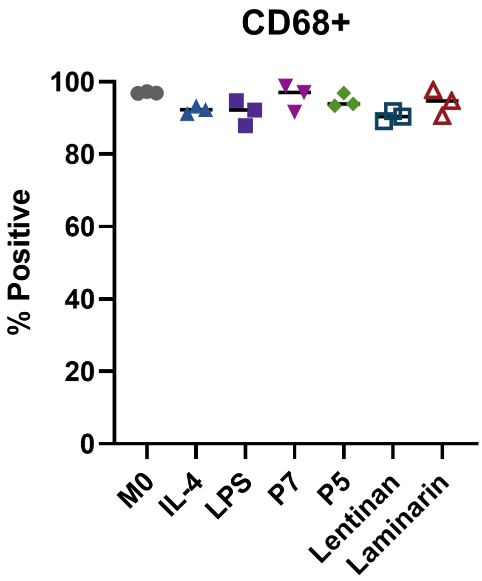


**Supplementary Figure 12:** Percentage of CD68+ cells from viable cells in culture after treatment with M0, LPS, IL-4, P7, P5, lentinan, or laminarin. Data are represented as mean percentage positive from three independent experiments (N = 3).

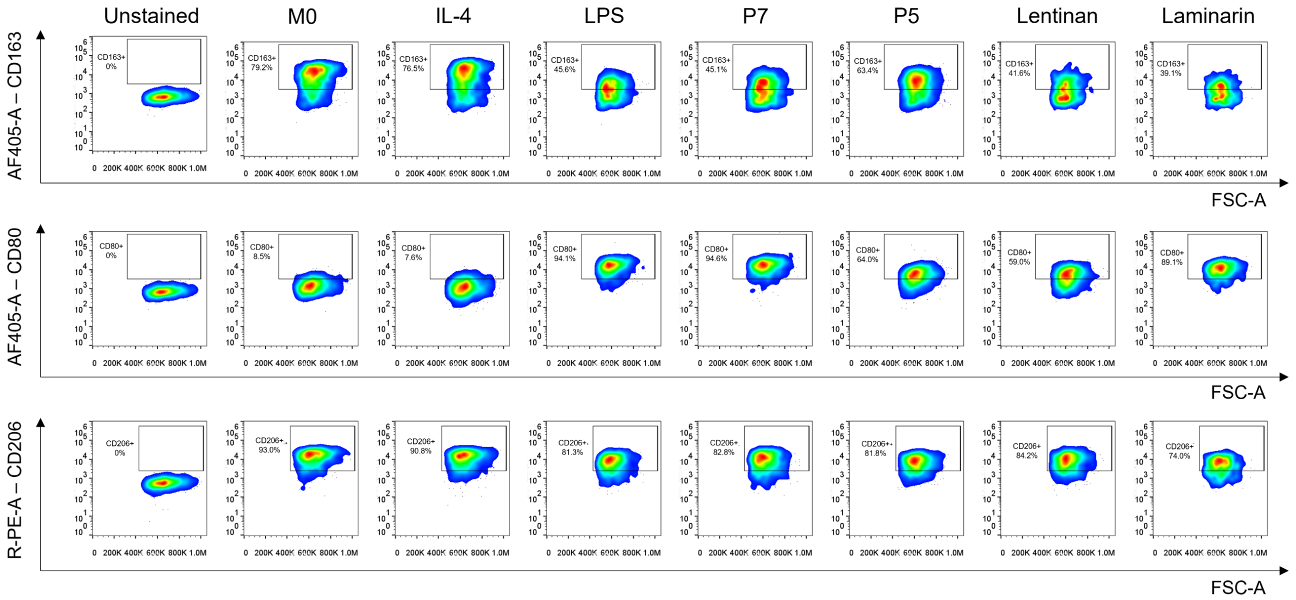


**Supplementary Figure 13:** Representative flow cytometry plots of CD68+ primary macrophages on day 8 post-M-CSF differentiation treated with nothing (M0), IL-4, LPS, P7, P5, lentinan, or laminarin. Each plot represents a separate trial.

$^{1}$H NMR spectrum of 2,3,4,2',3',4',6'-hepta-*O*-acetyl-D-gentiobiosyl bromide (**2**)

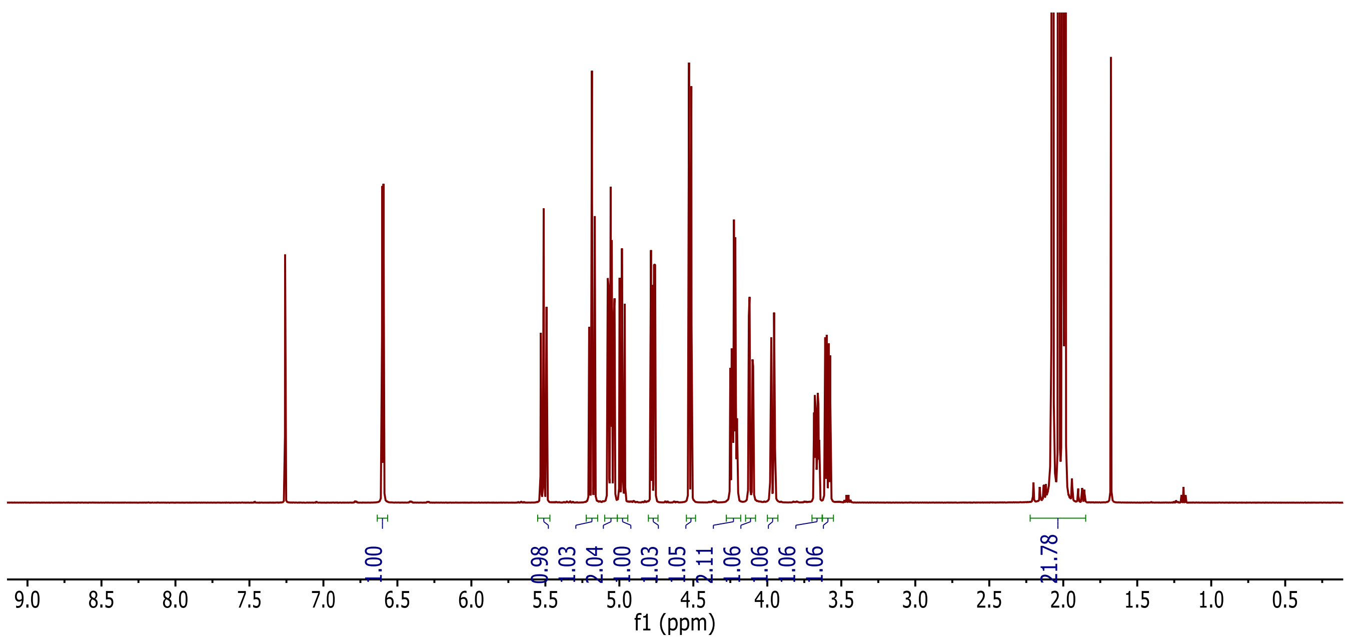

$^{1}$H NMR spectrum of 3,4,2',3',4',6'-hexo-*O*-benzyl-D-gentiobial (**3**)

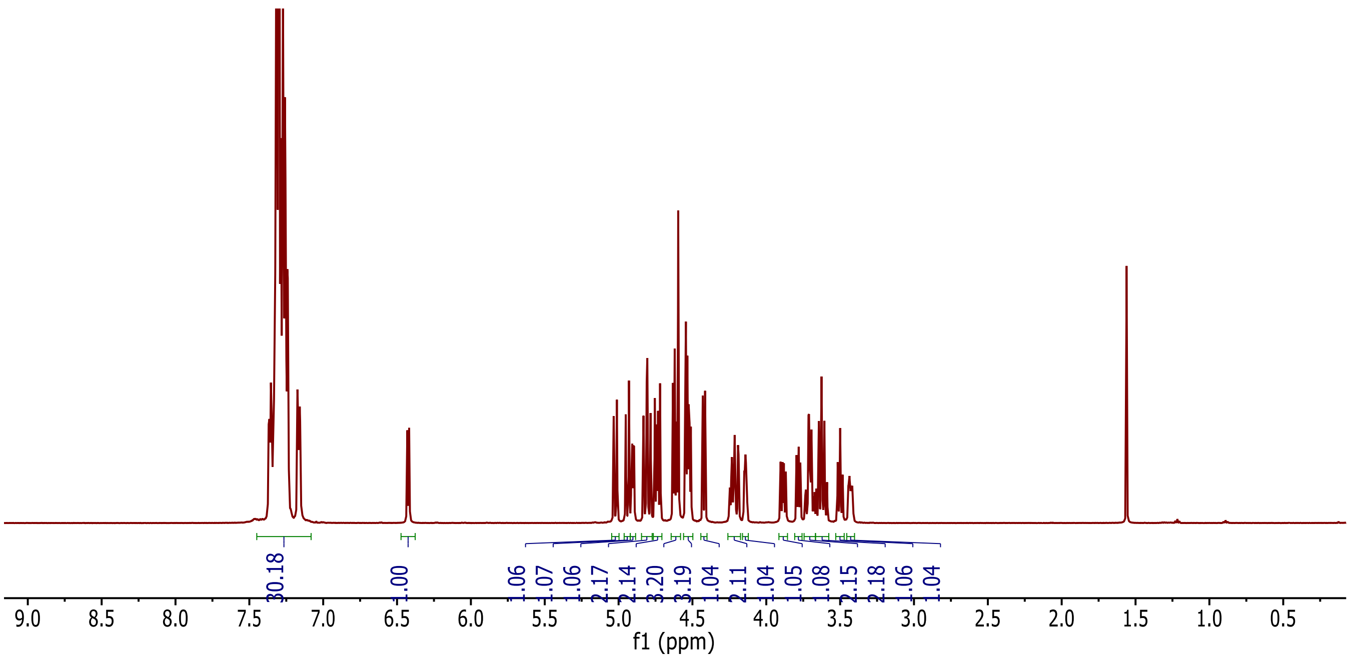

$^{1}$H NMR spectrum of Gen-lactam

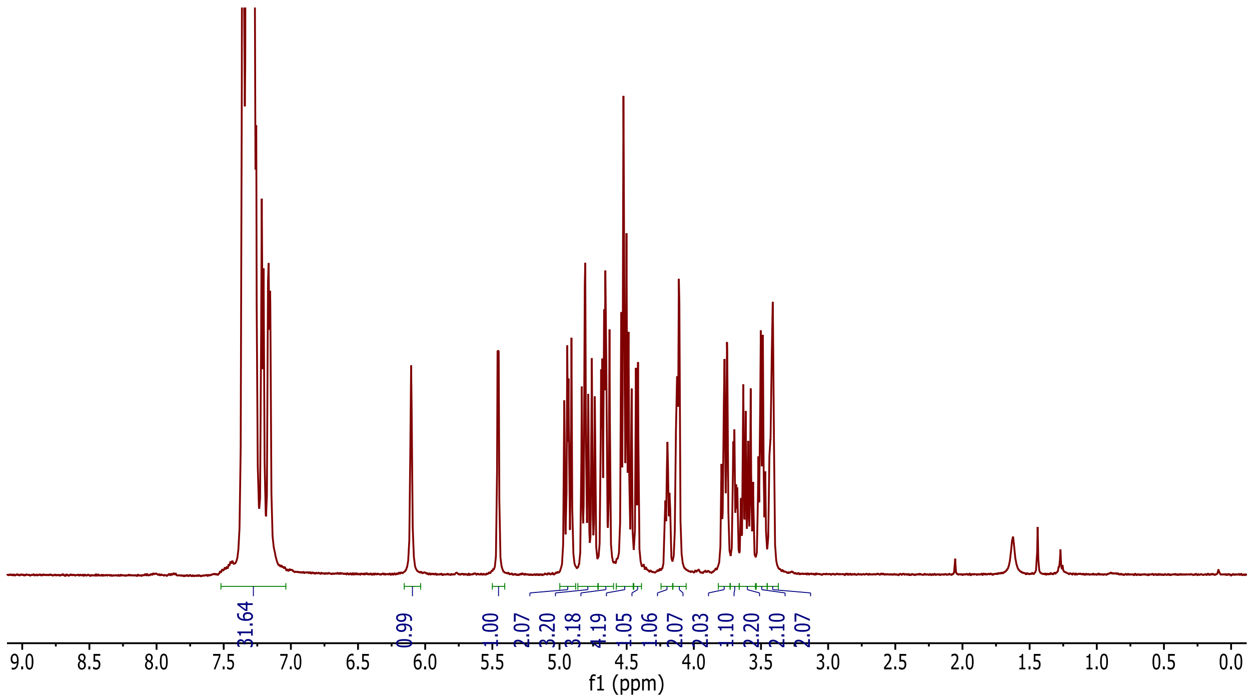

COSY NMR spectrum of Gen-lactam

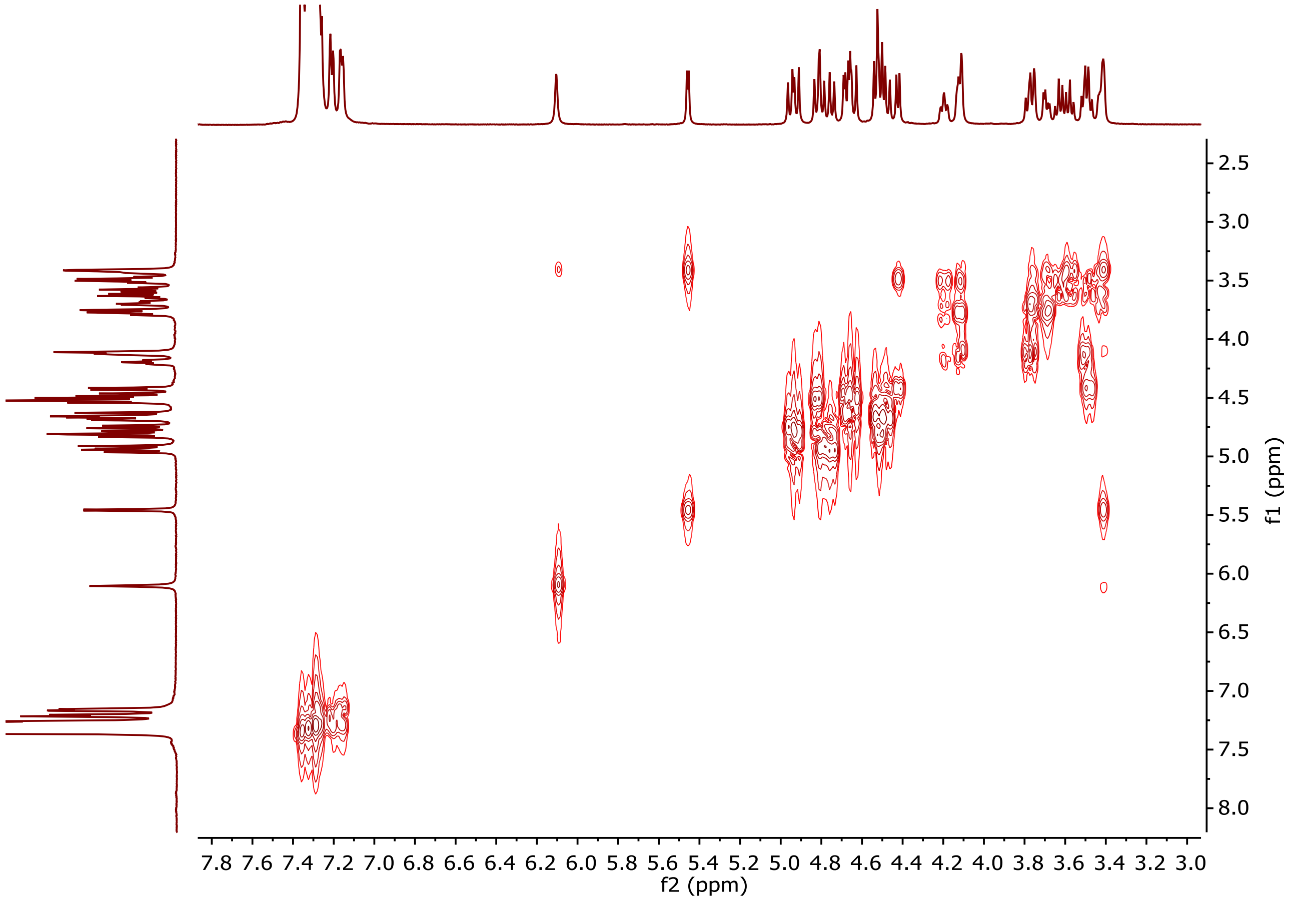


$^{13}$C NMR spectrum of Gen-lactam

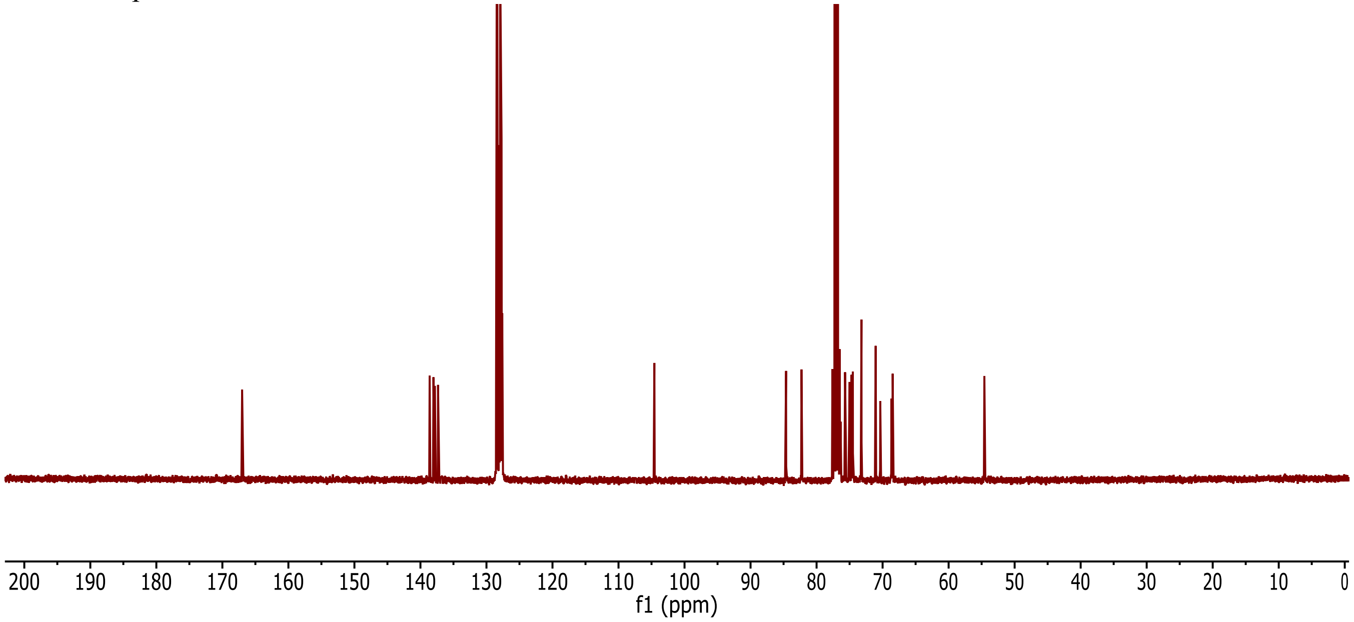

HSQC spectrum of Gen-lactam

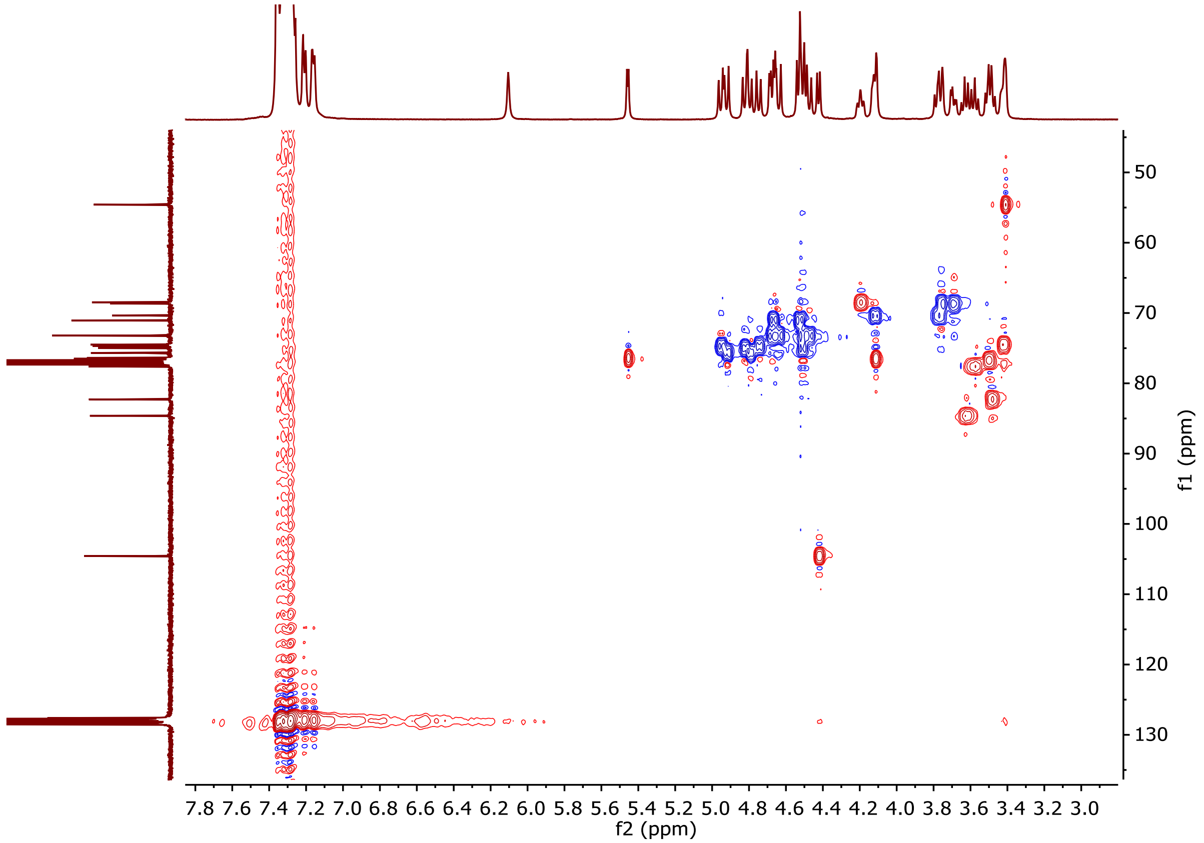


HMBC spectrum of Gen-lactam

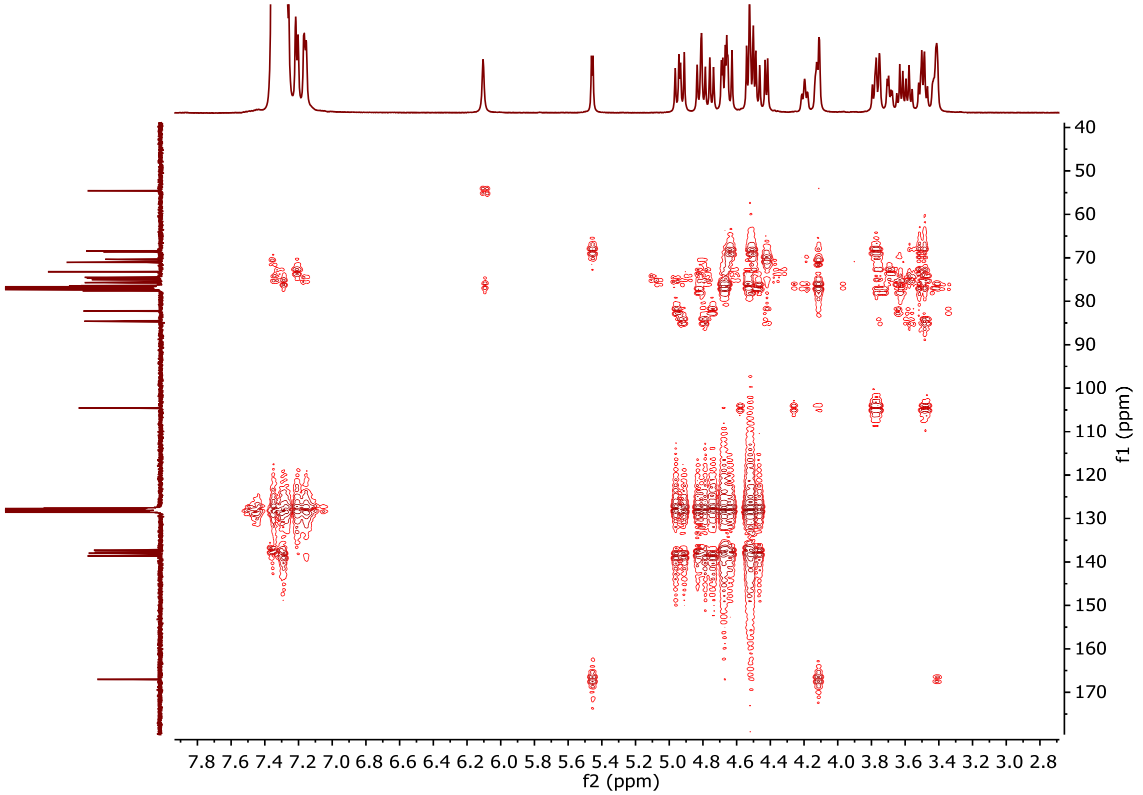

FTIR spectrum of Gen-lactam

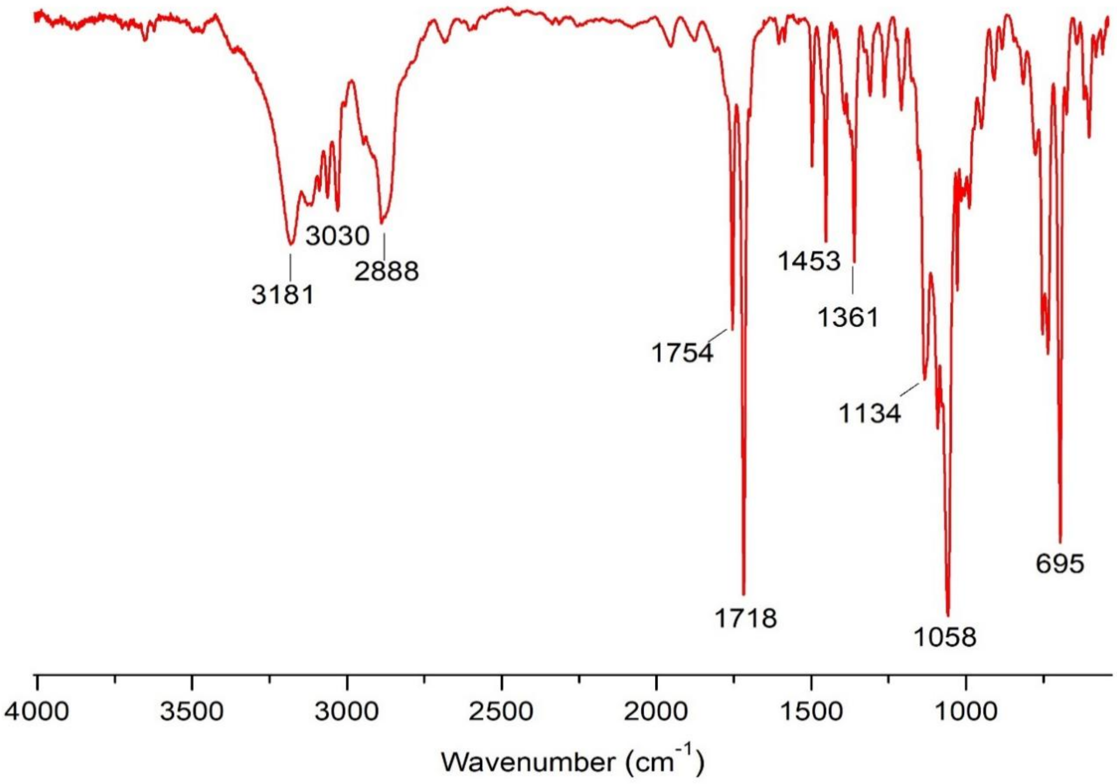


$^1$H NMR spectrum of P1'

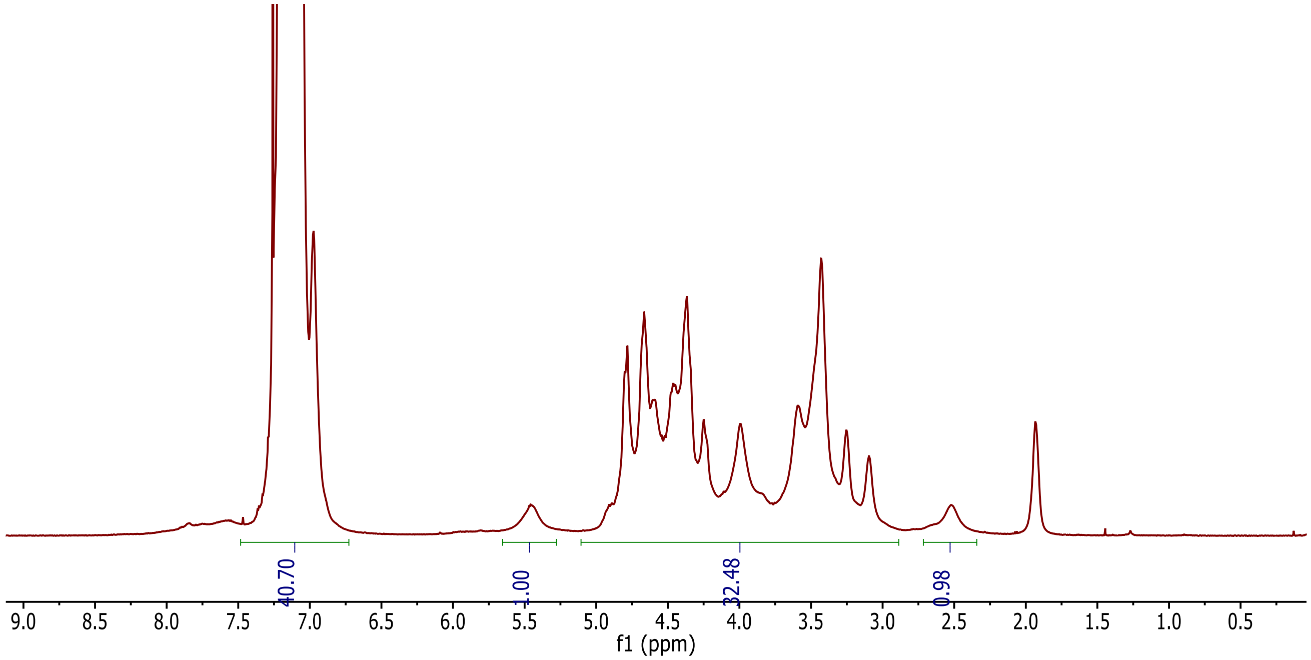

$^{1}H$ NMR spectrum of P2'

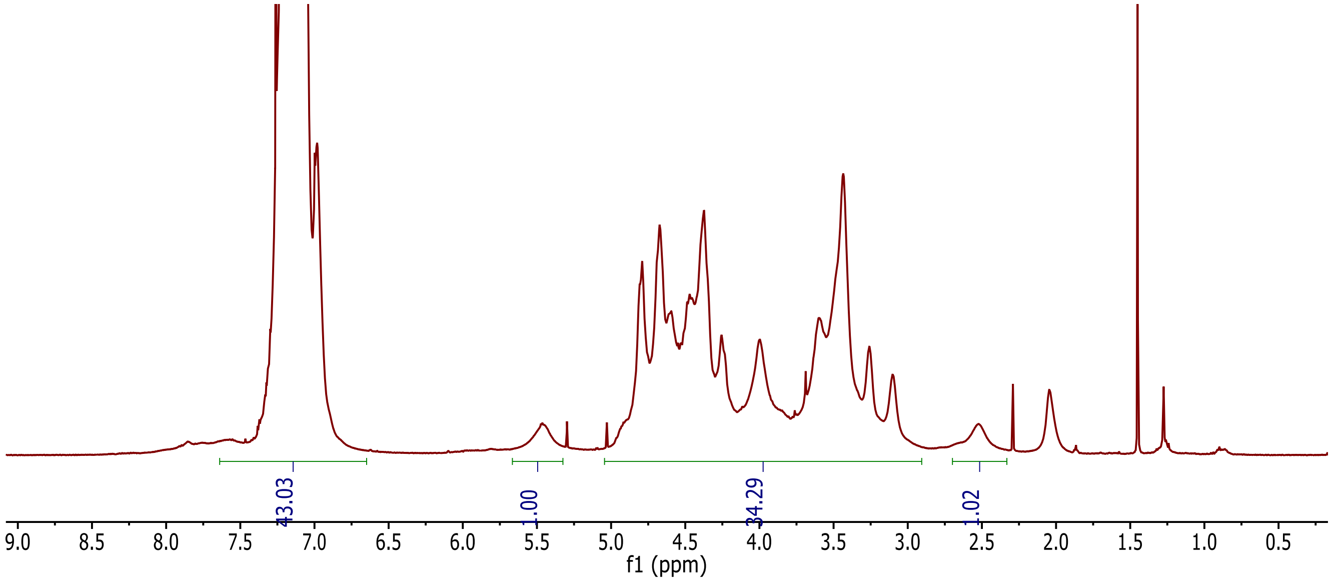


$^{1}H$ NMR spectrum of P3'

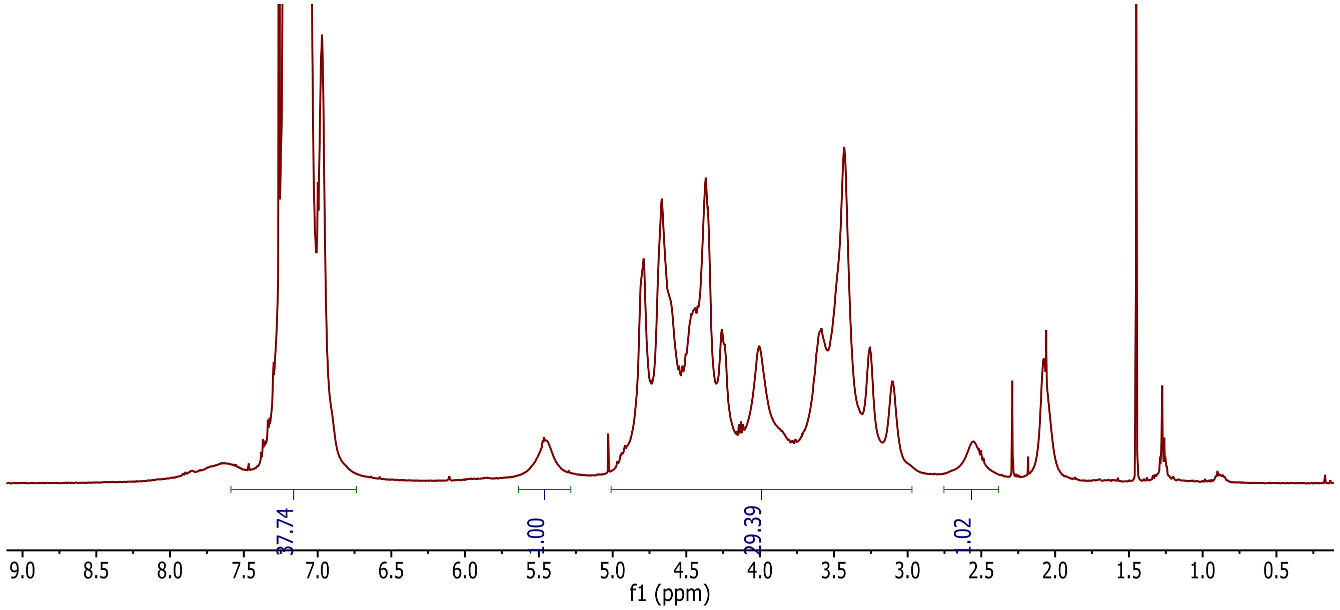

$^{1}$H NMR spectrum of P4'

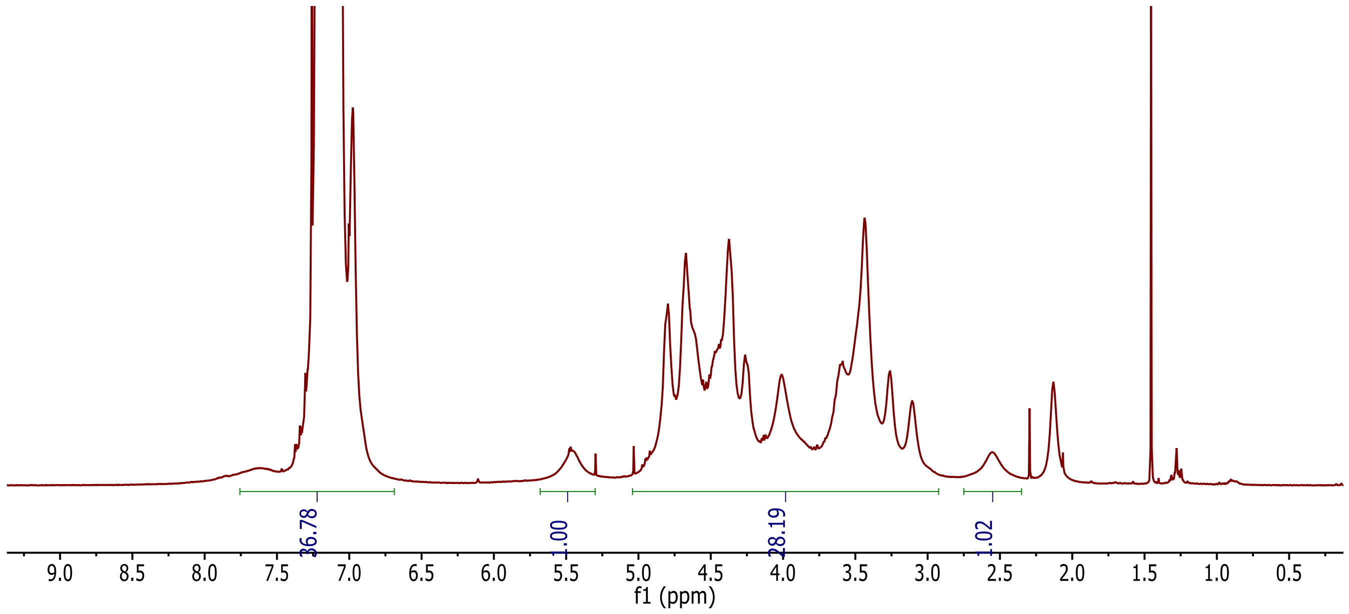


$^{1}$H NMR spectrum of P1

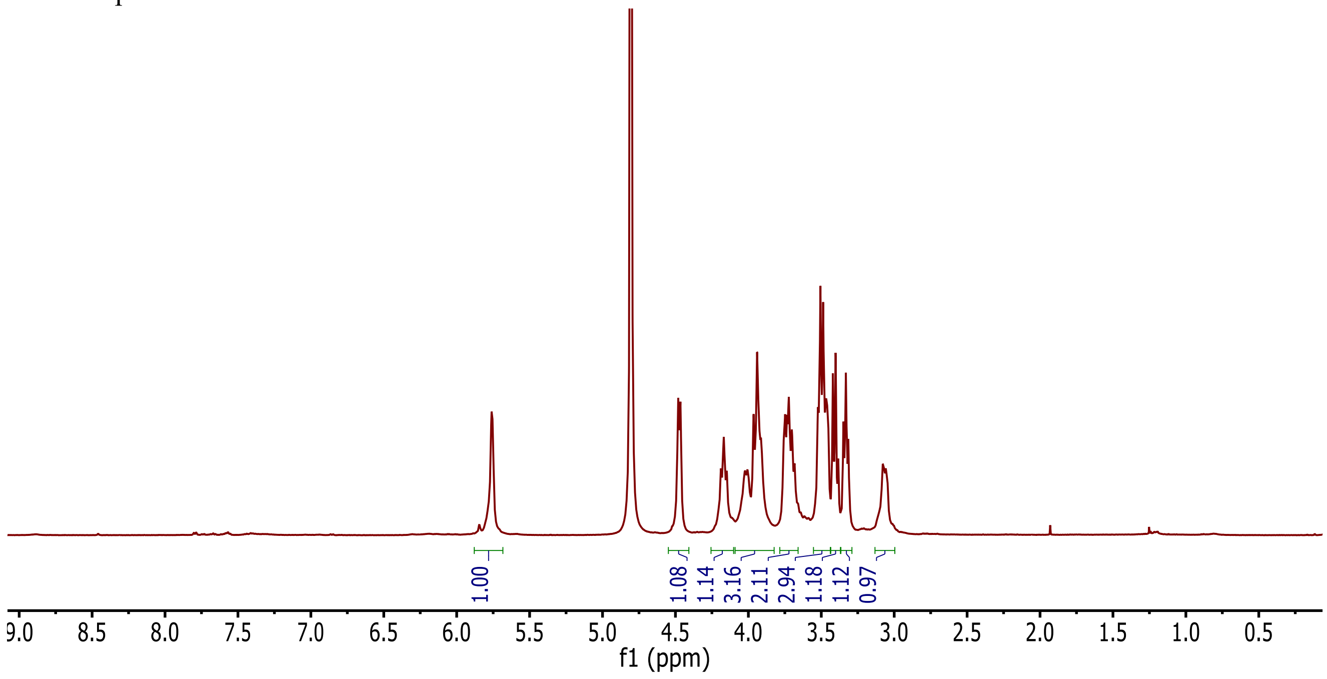

COSY spectrum of P1

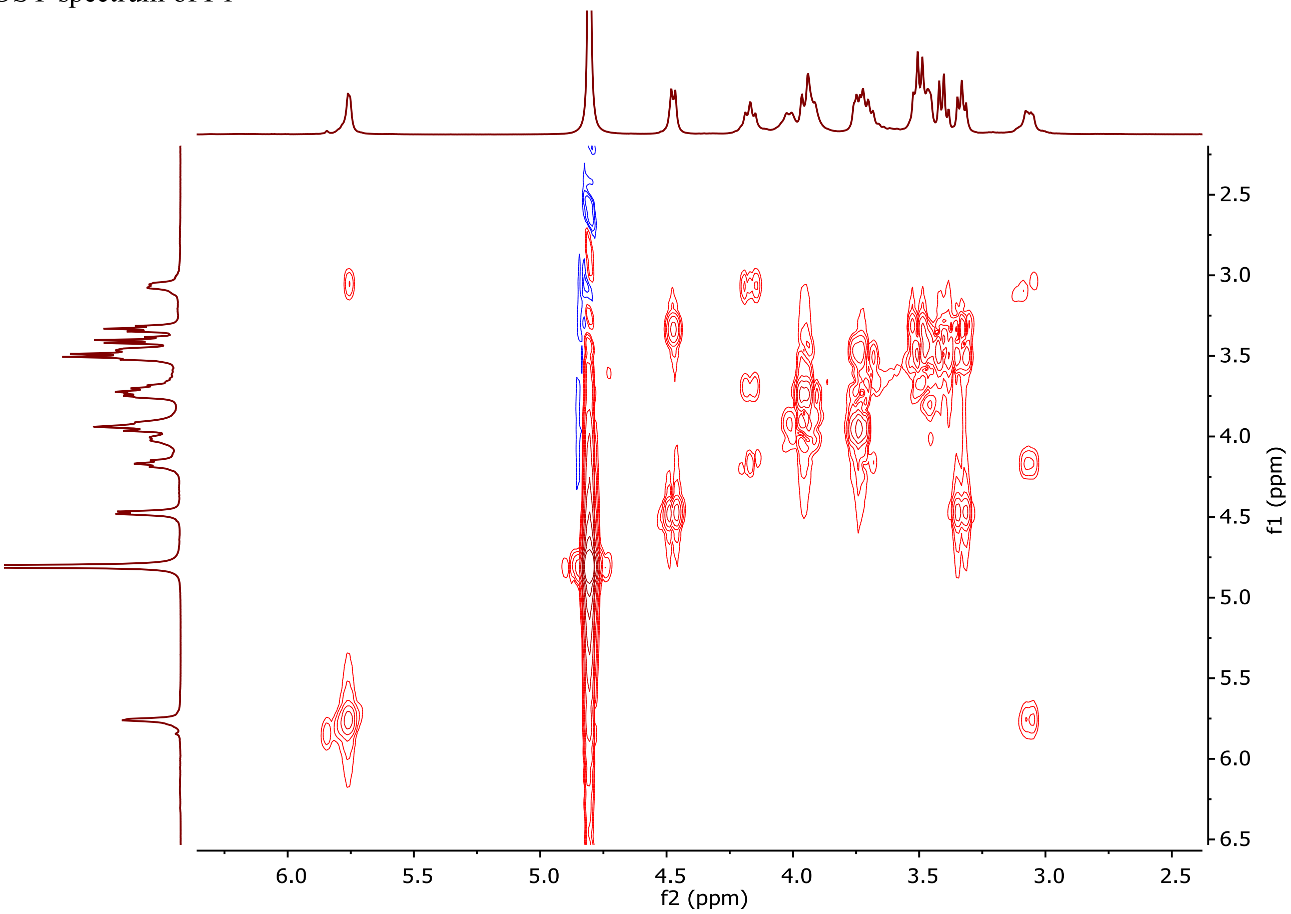


$^{13}C$ NMR spectrum of P1

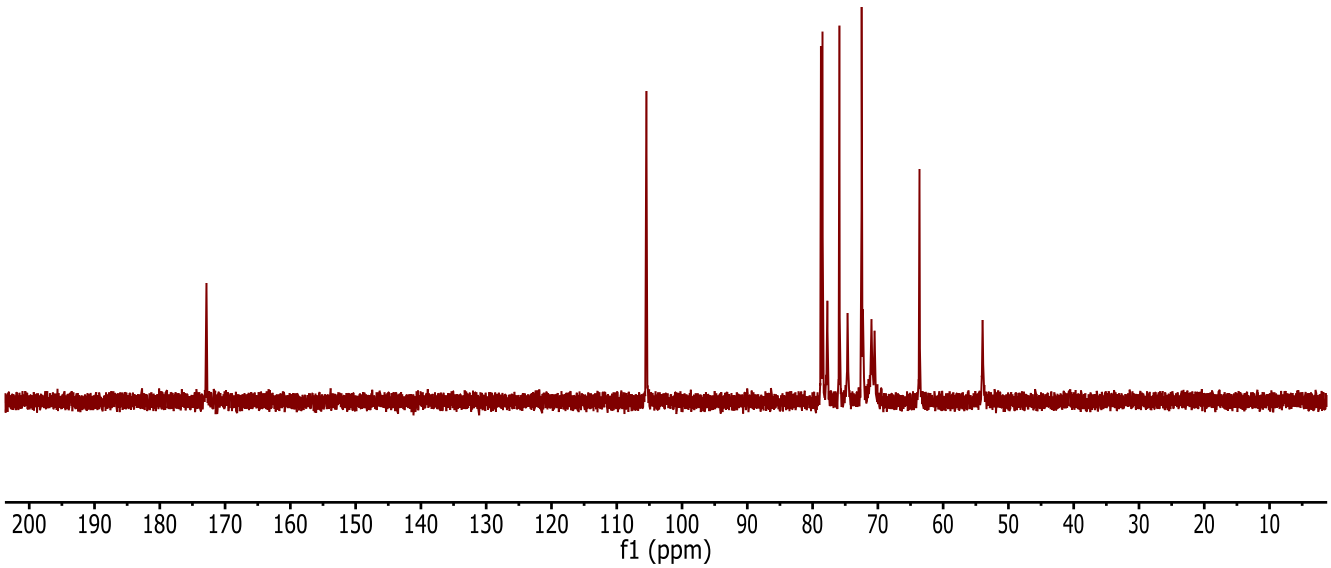

HSQC spectrum of P1

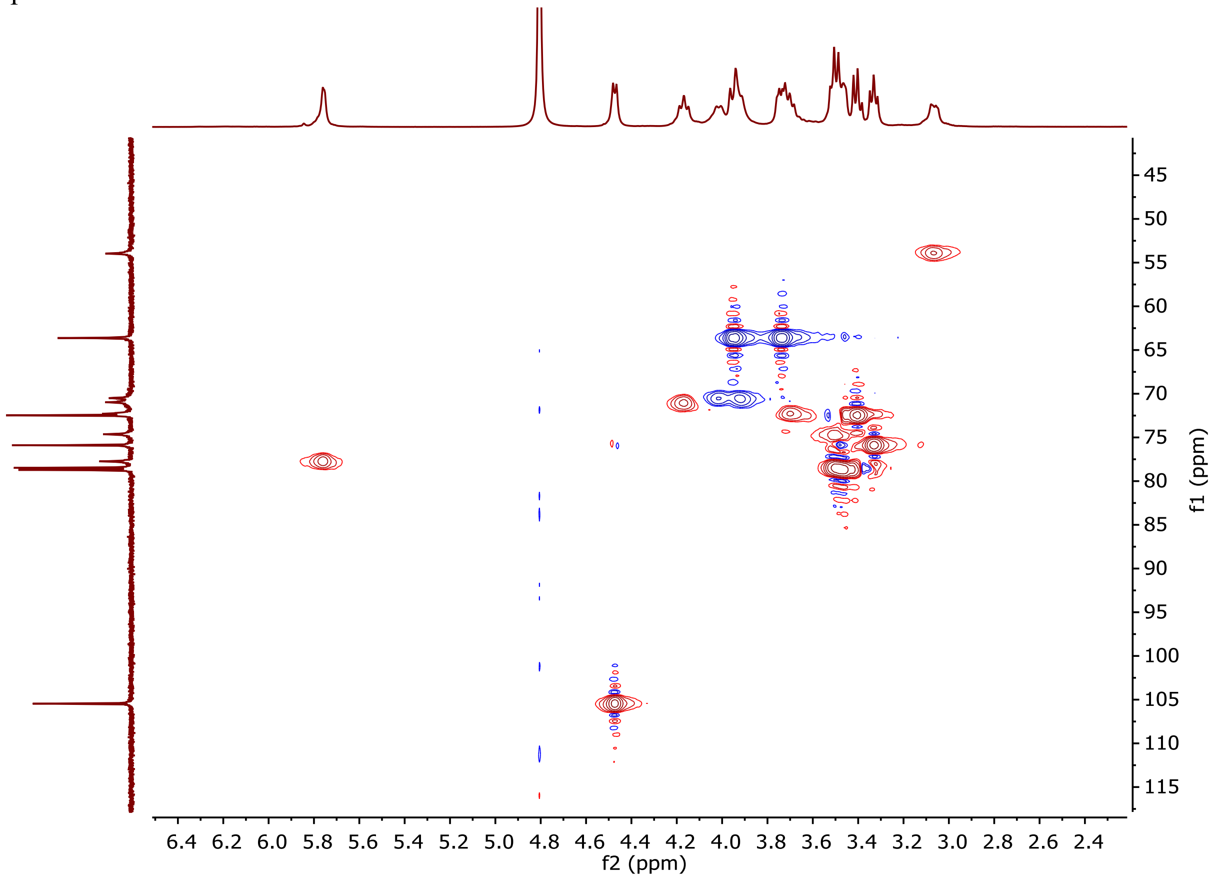


$^{1}H$ NMR spectrum of P2

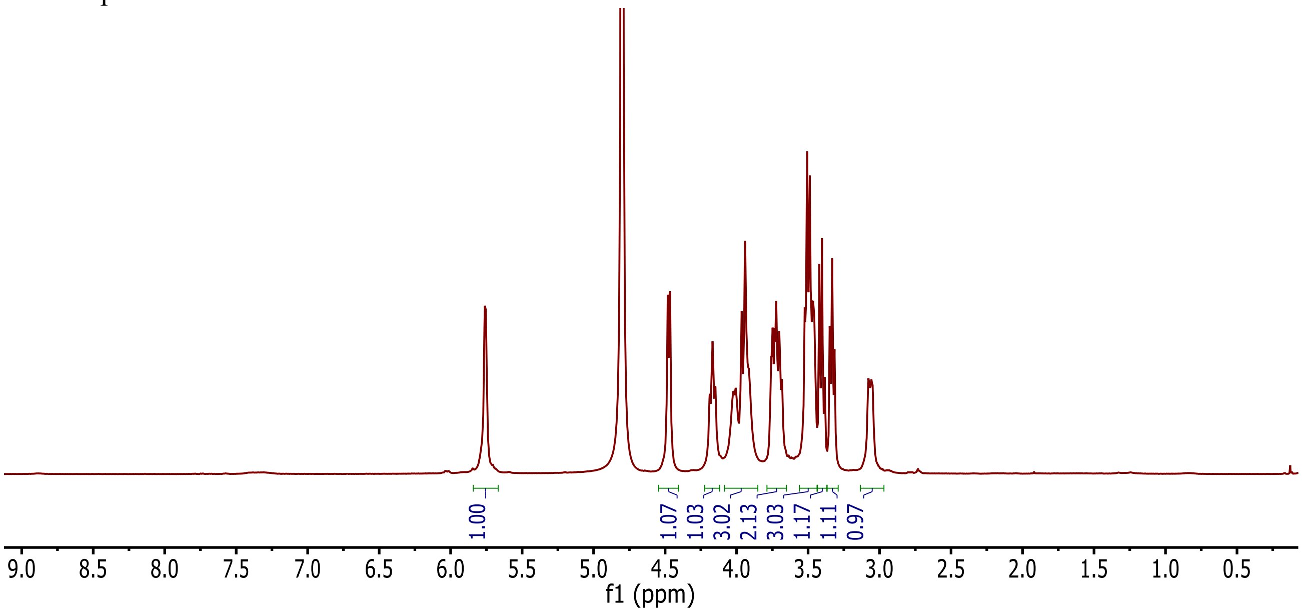

COSY spectrum of P2

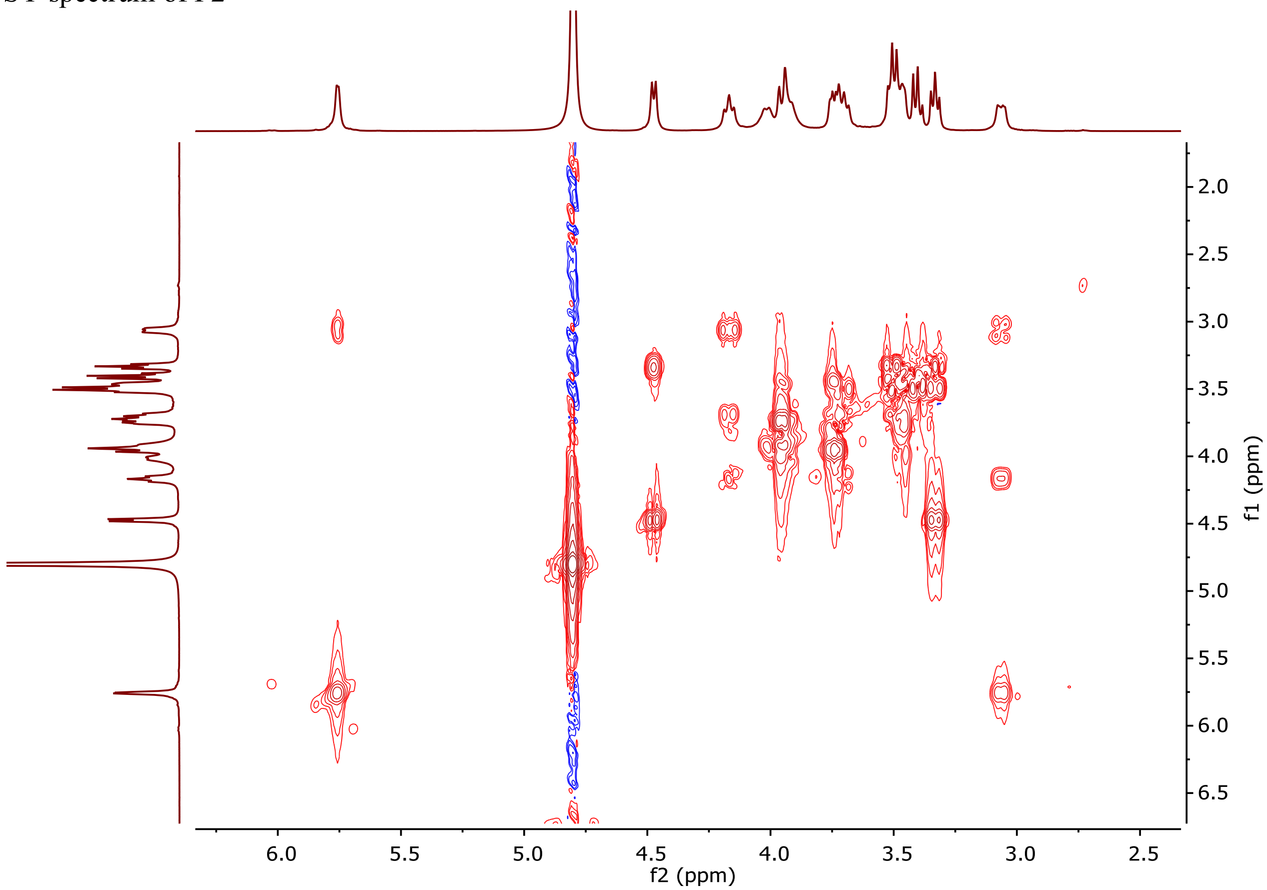


$^{13}$C NMR spectrum of P2

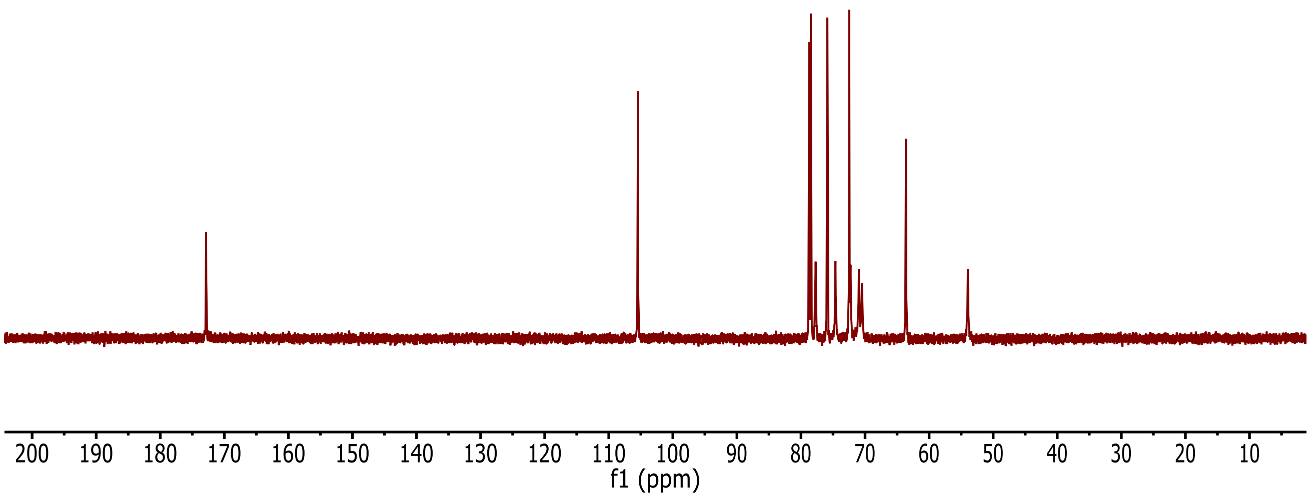

HSQC spectrum of P2

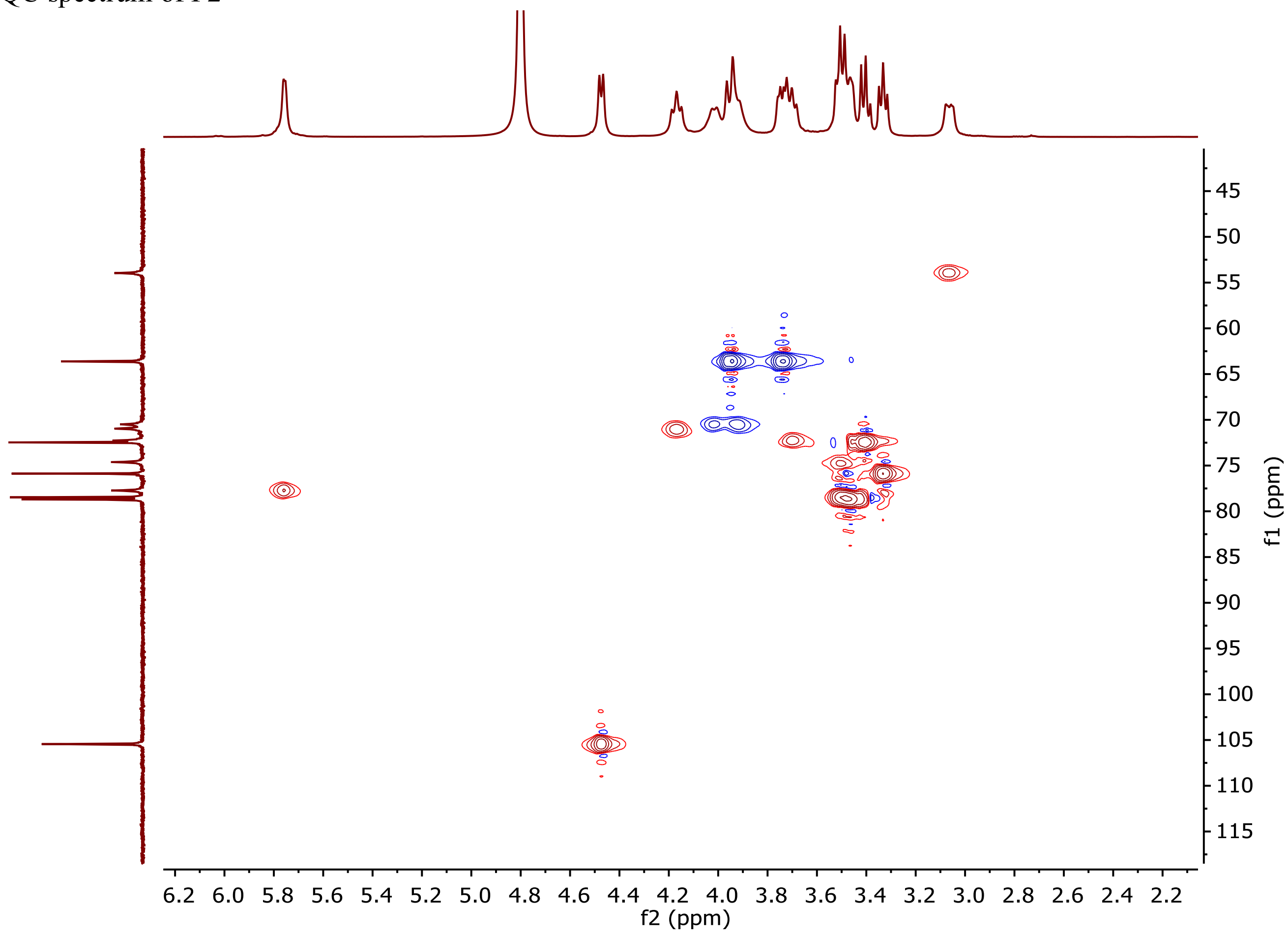


$^{1}$H NMR spectrum of P3

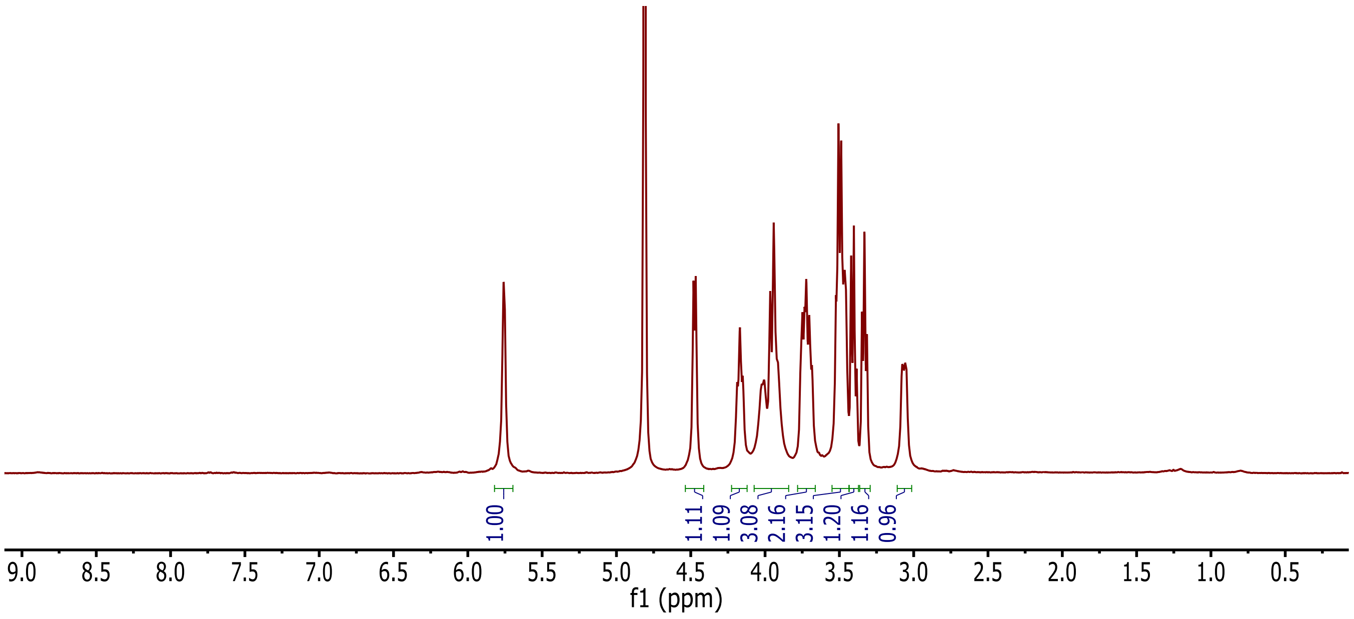

COSY spectrum of P3

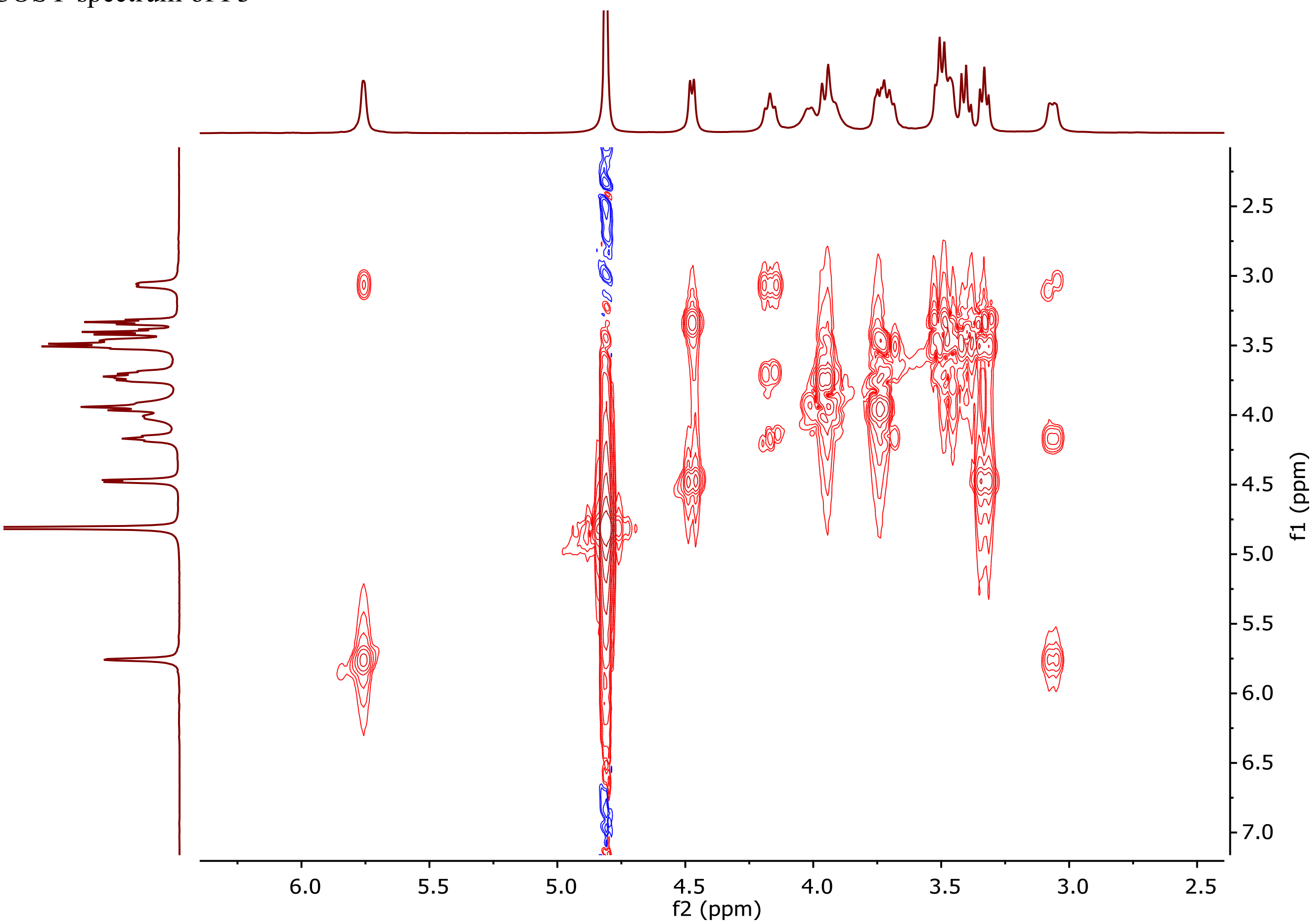


$^{13}C$ NMR spectrum of P3

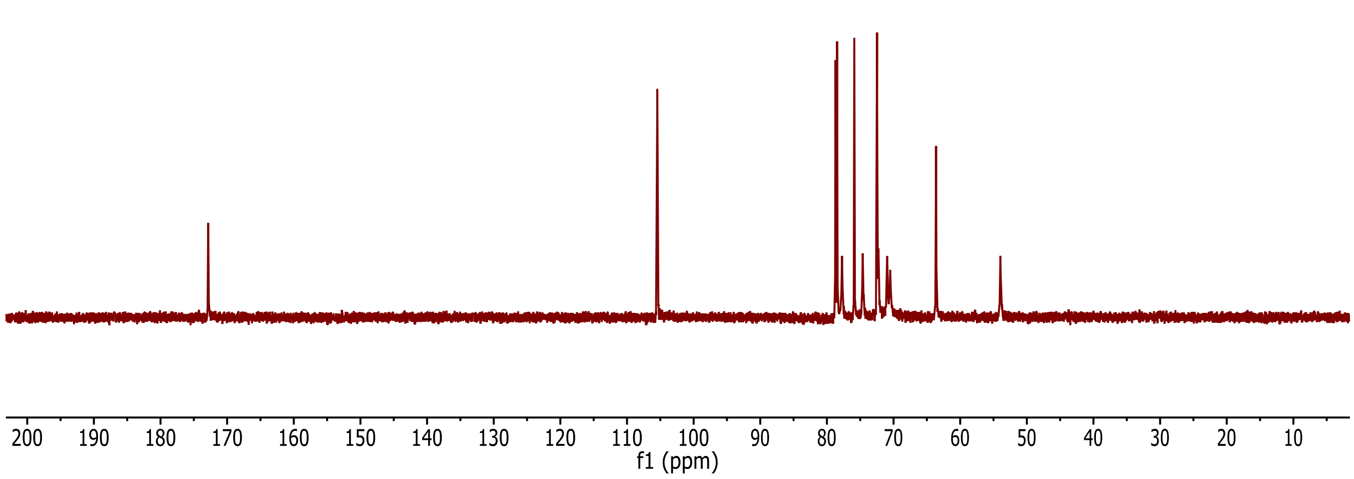

HSQC spectrum of P3

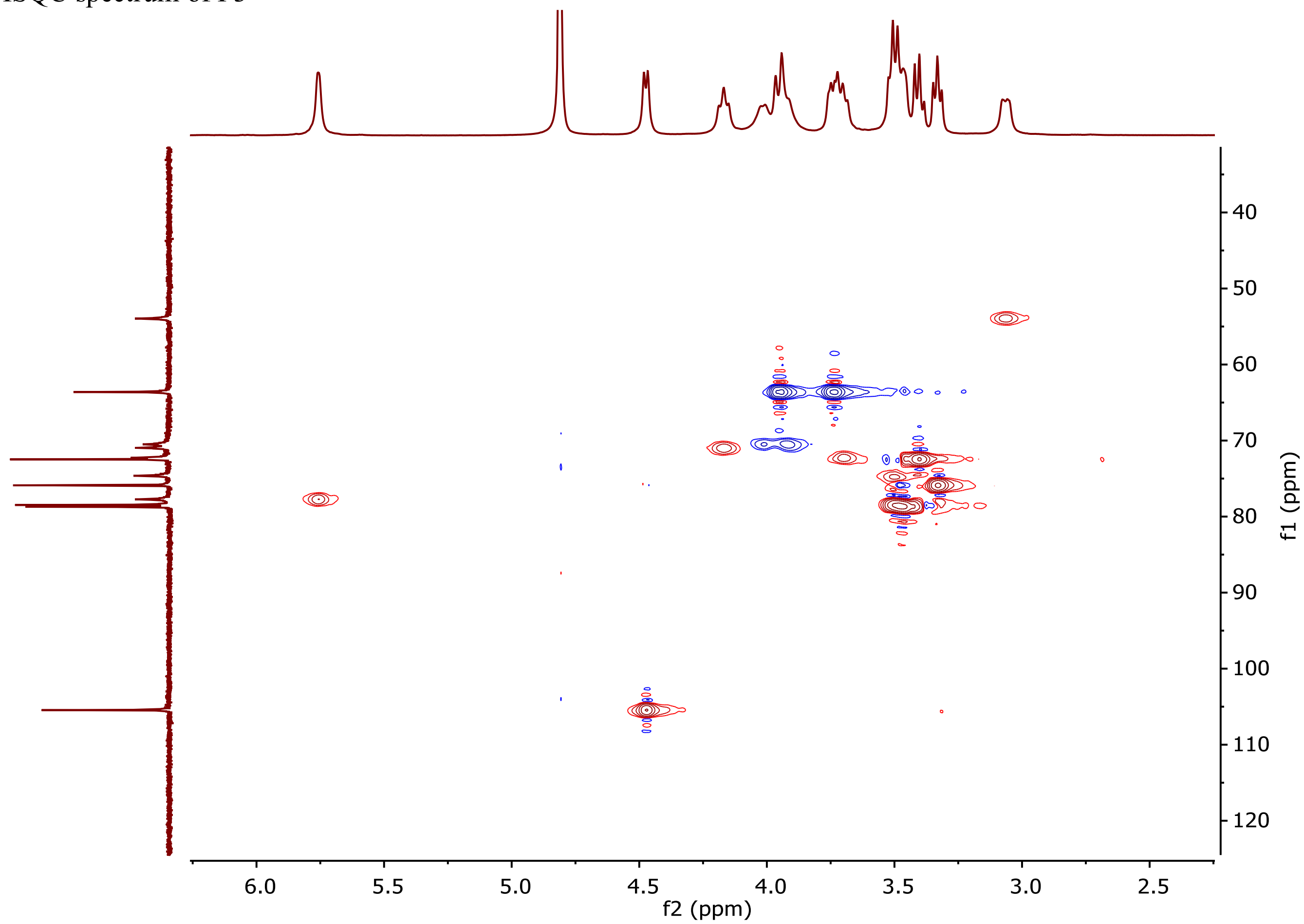


$^{1}$H NMR spectrum of P4

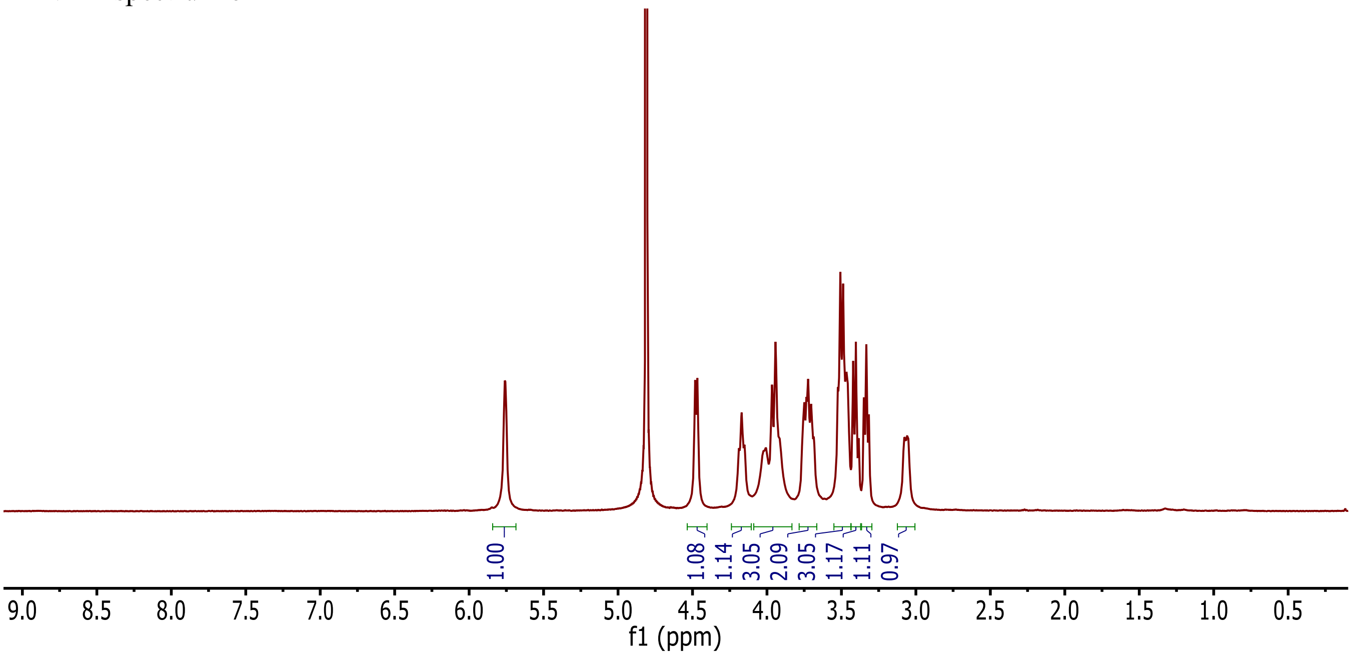

COSY spectrum of P4

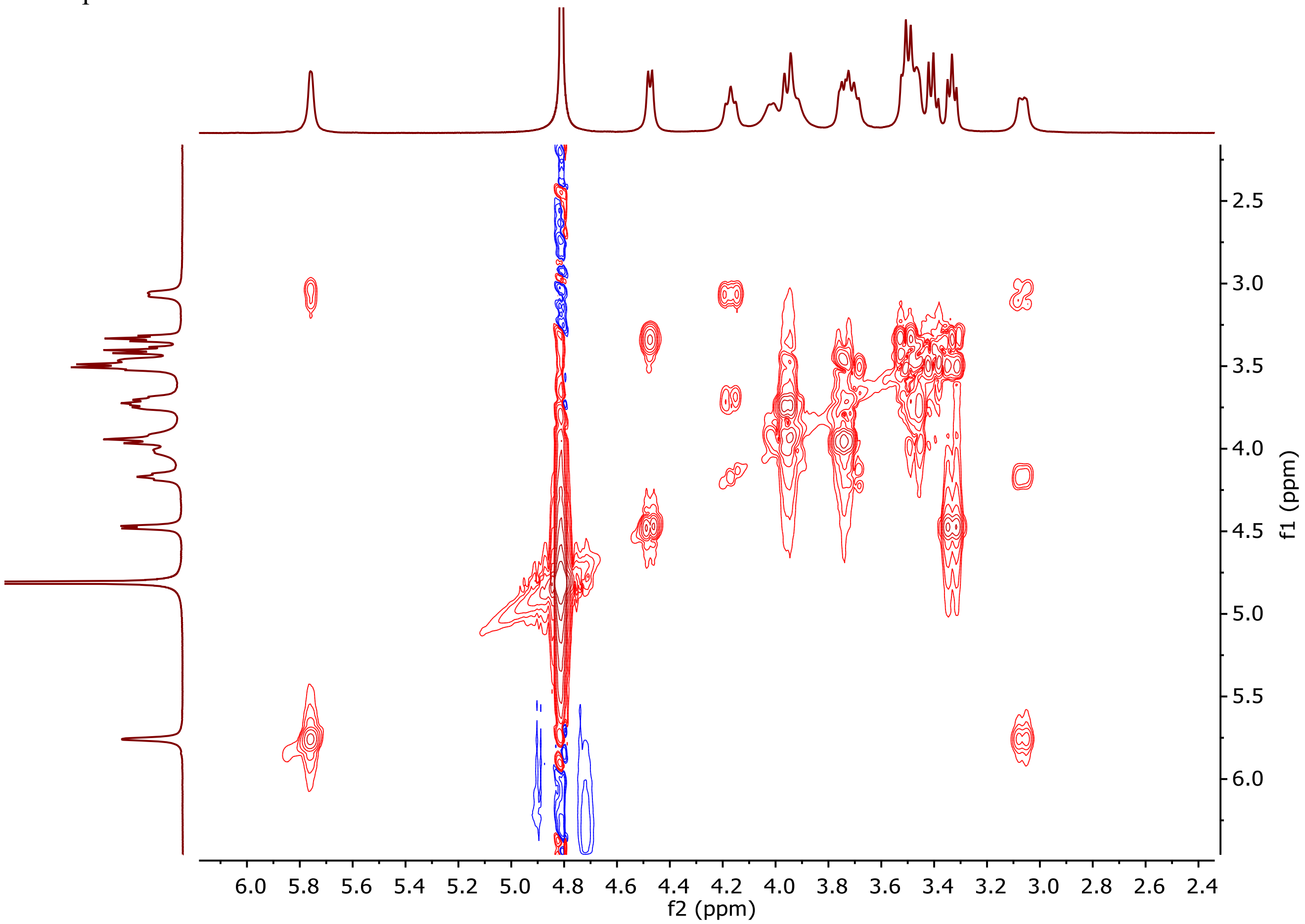


$^{13}C$ NMR spectrum of P4

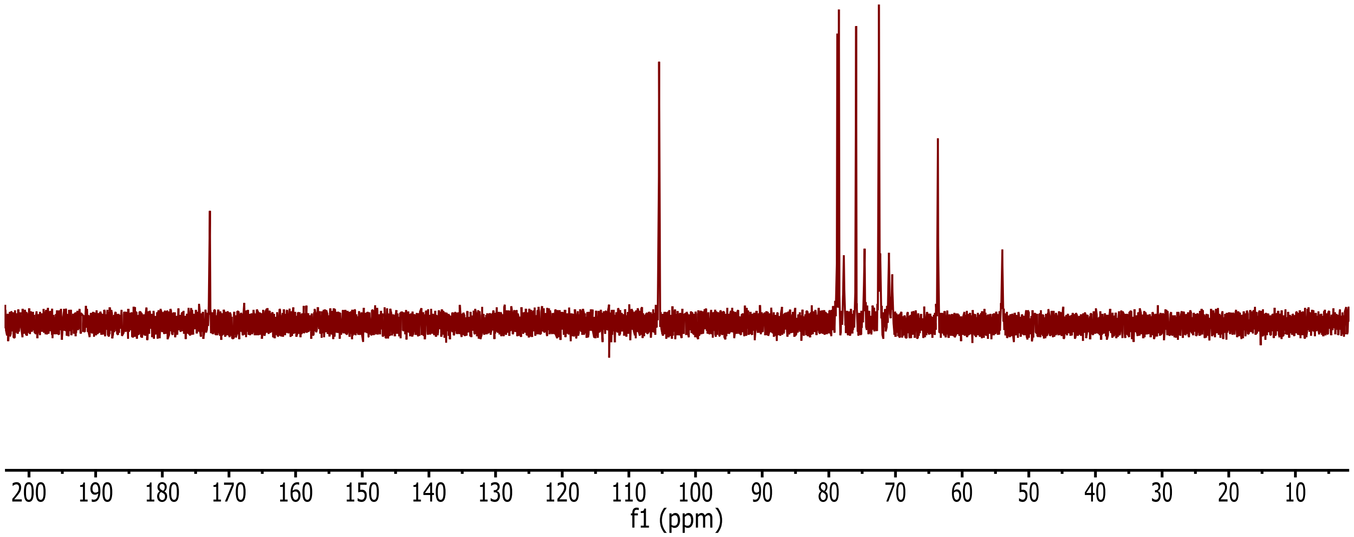

HSQC spectrum of P4

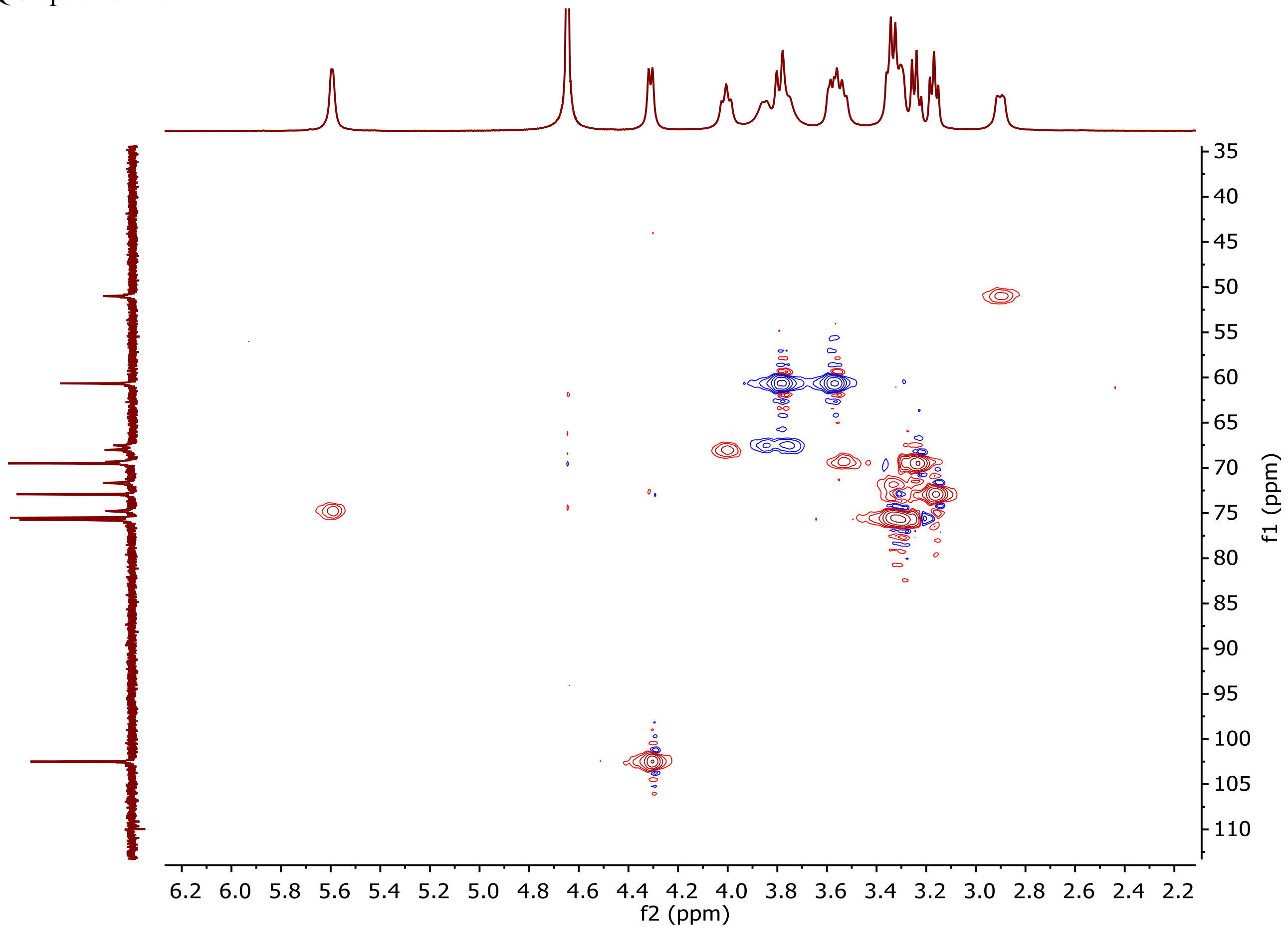


$^1$H NMR spectrum of P5

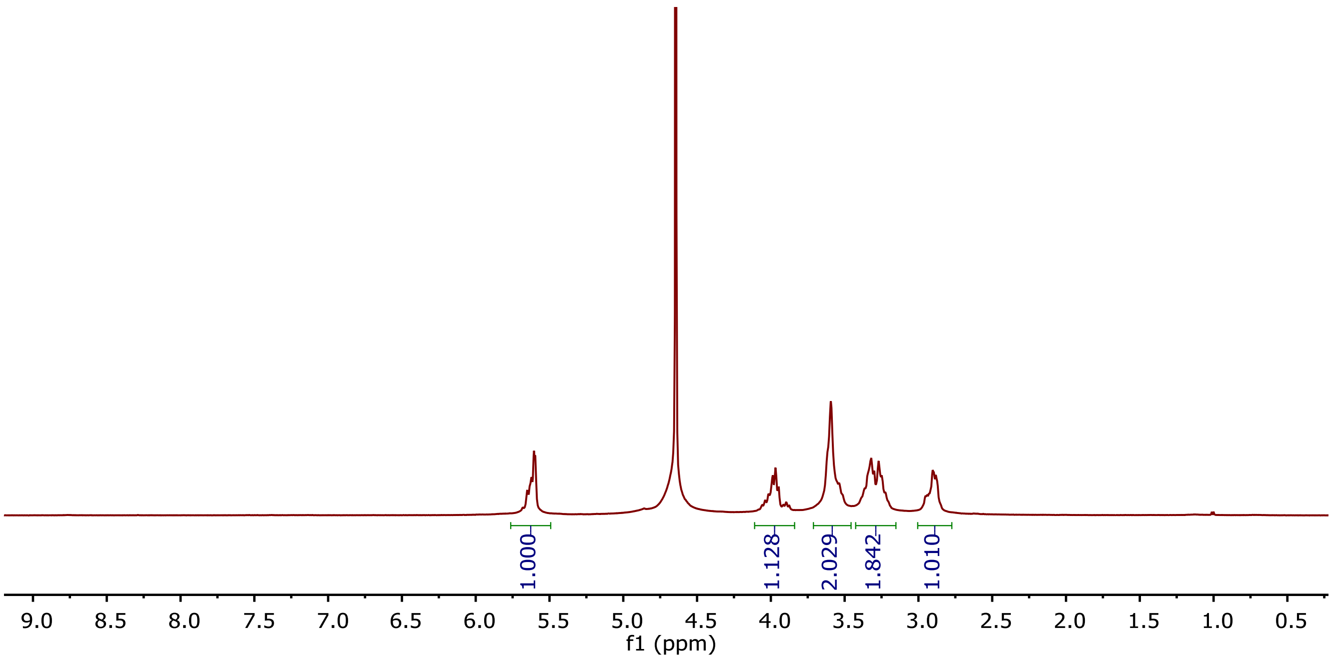

<sup></sup>

$^{1}$H NMR spectrum of P6

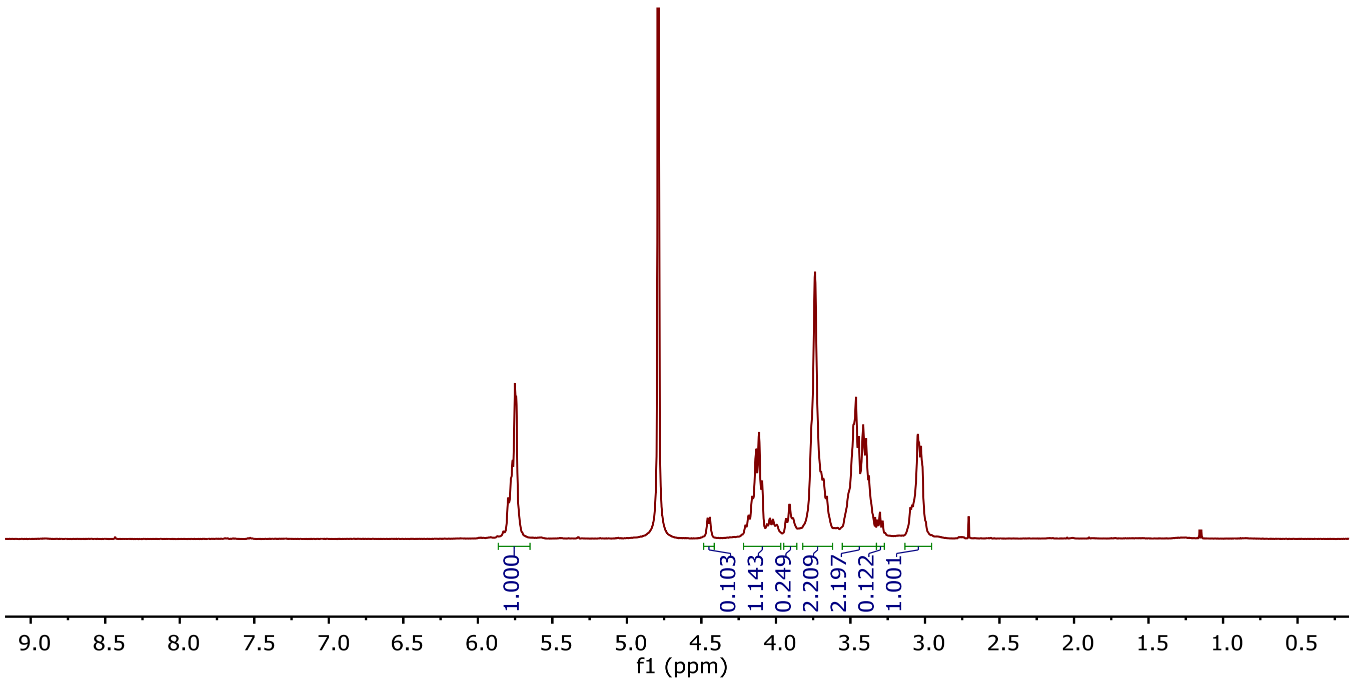


$^{1}$H NMR spectrum of P7

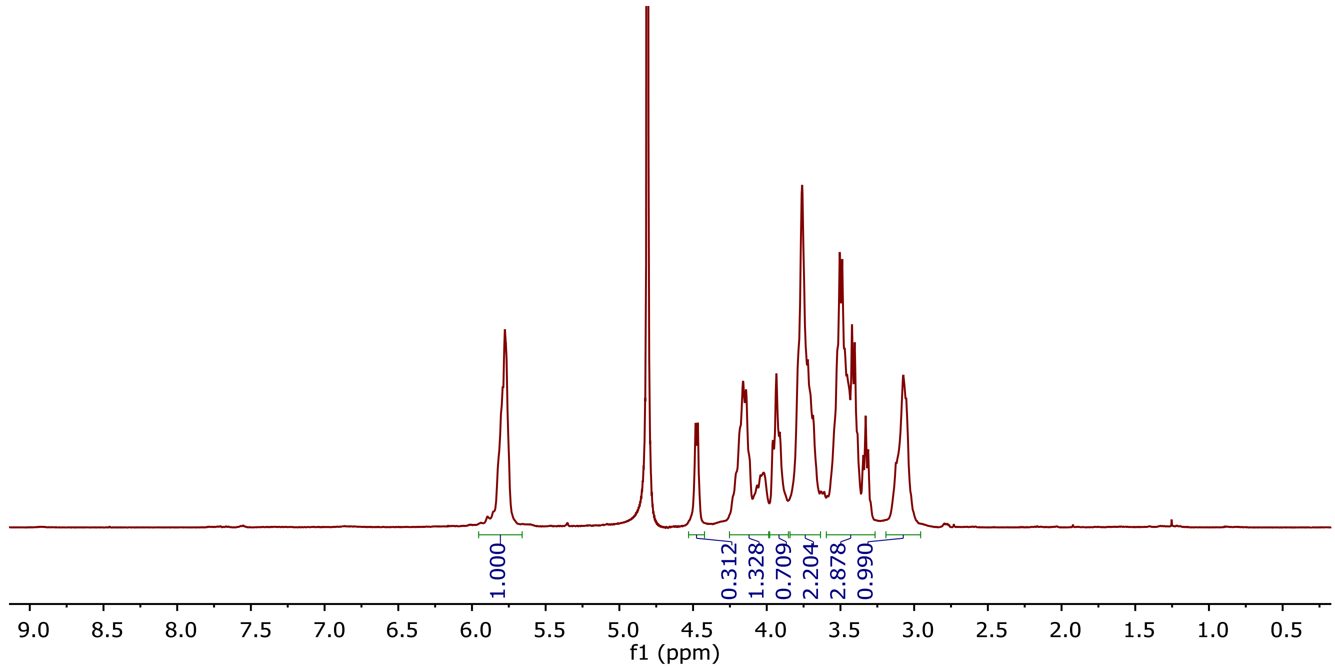

$^{1}$H NMR spectrum of P8

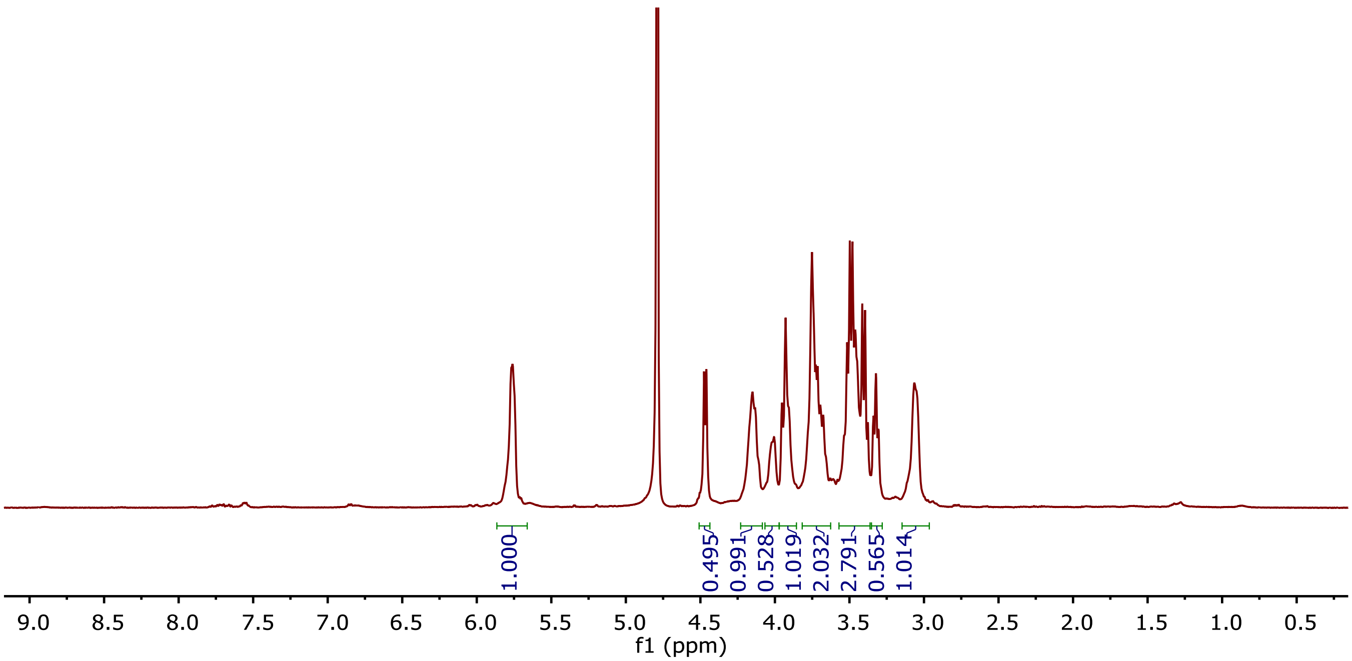


$^{1}$H NMR spectrum of P9

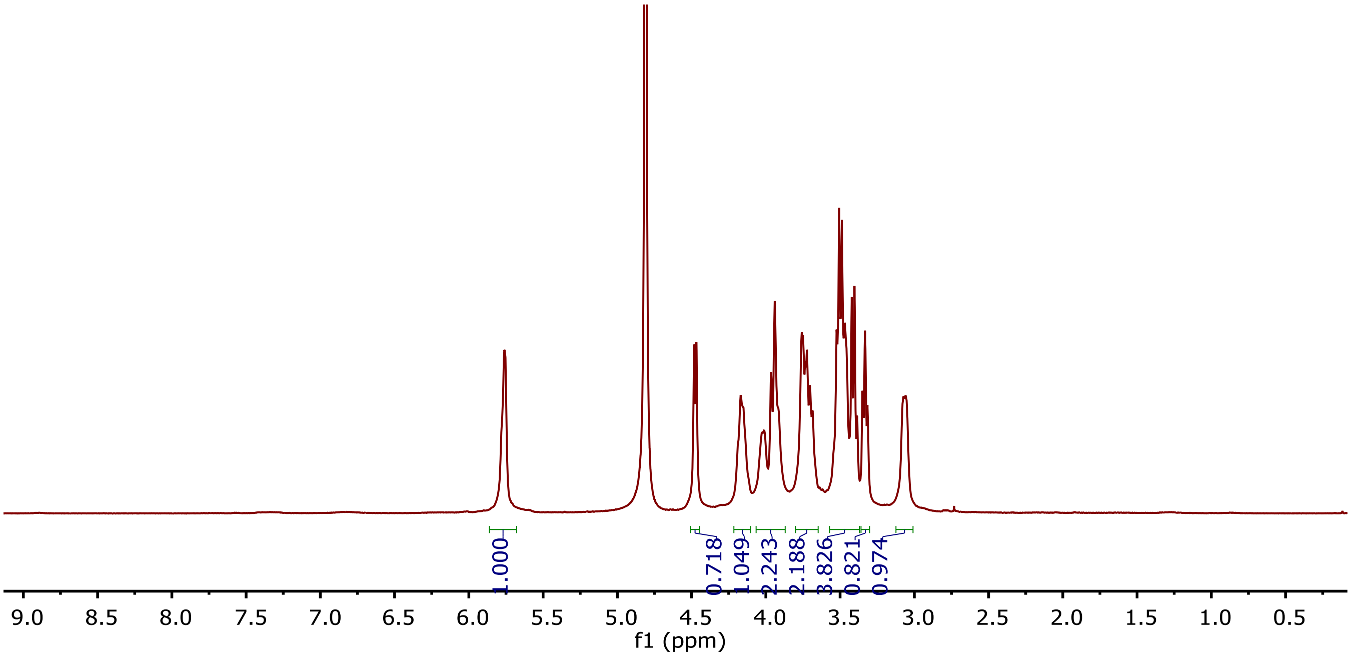

<sup></sup>

$^{1}$H NMR spectrum of P10

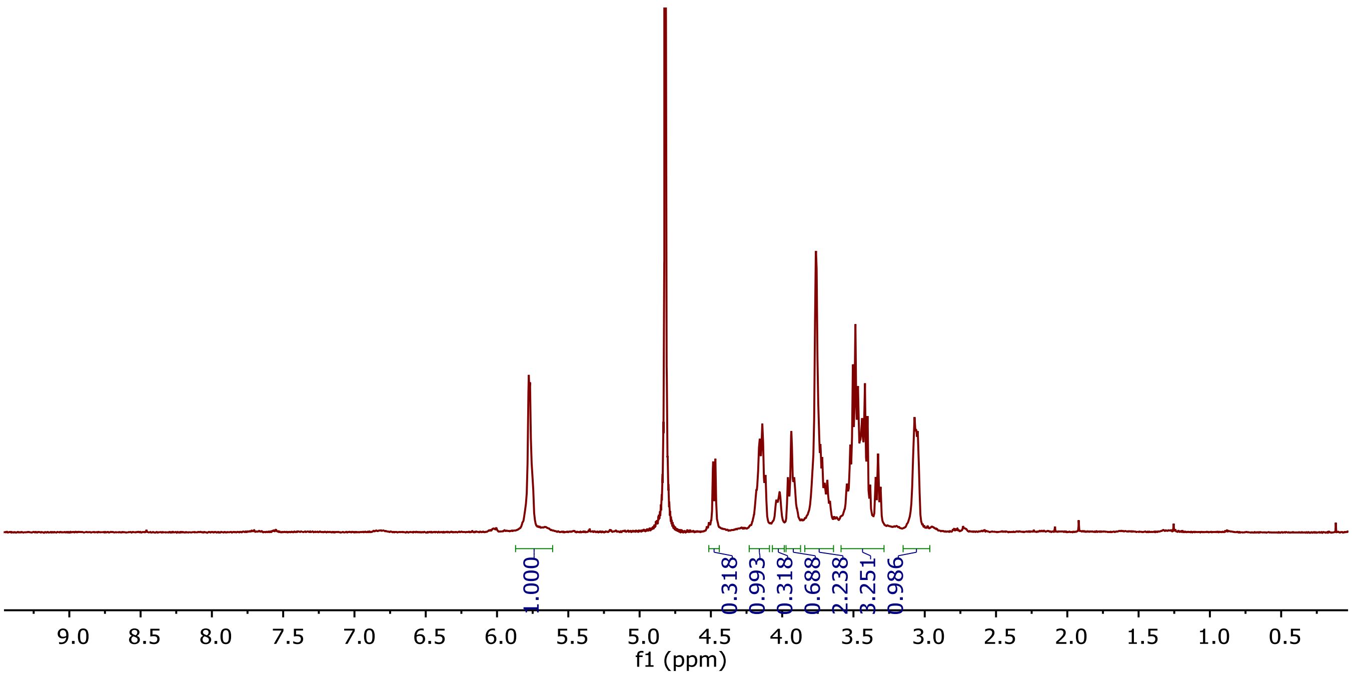


$^{1}$H NMR spectrum of P11

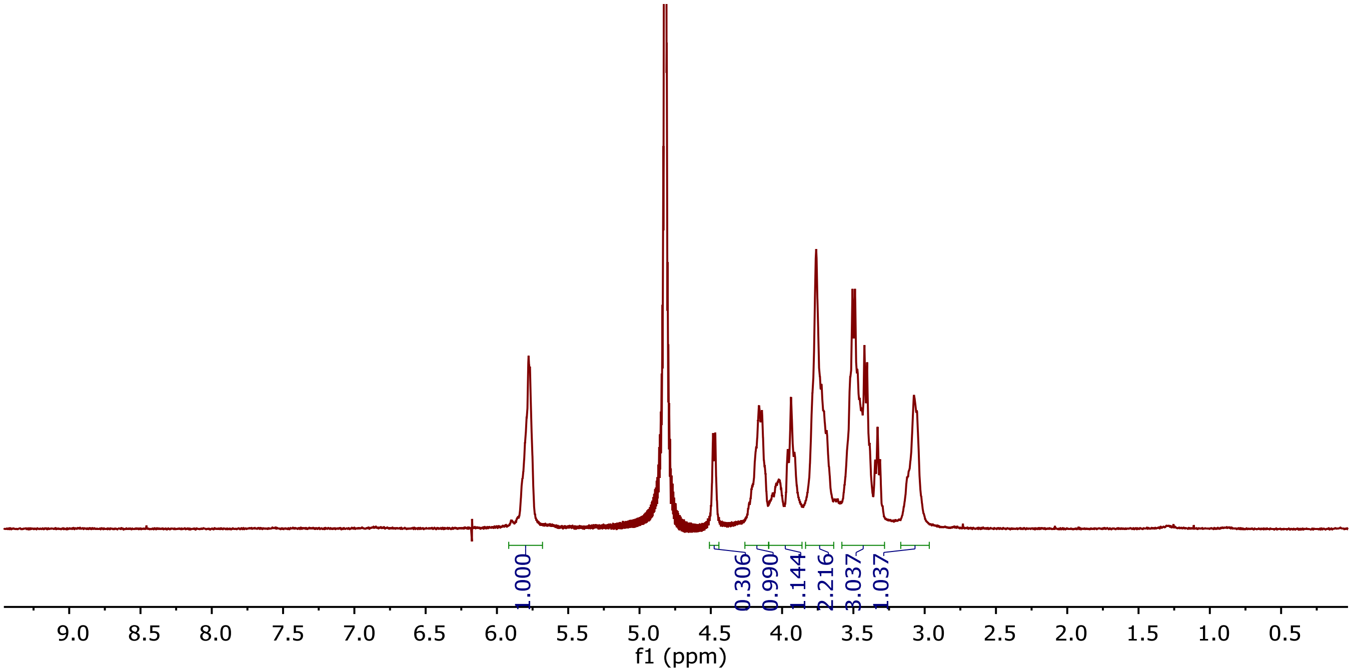

$^1H$ NMR spectrum of P12

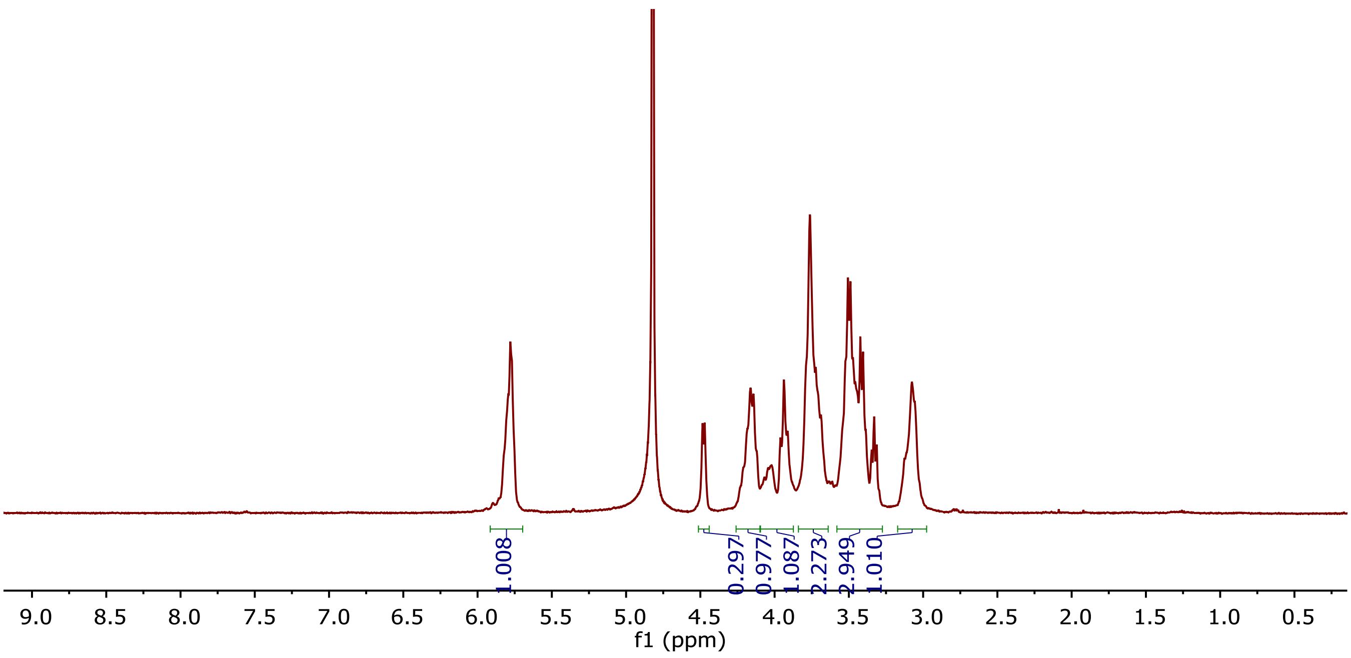


$^1H$ NMR spectrum of P13

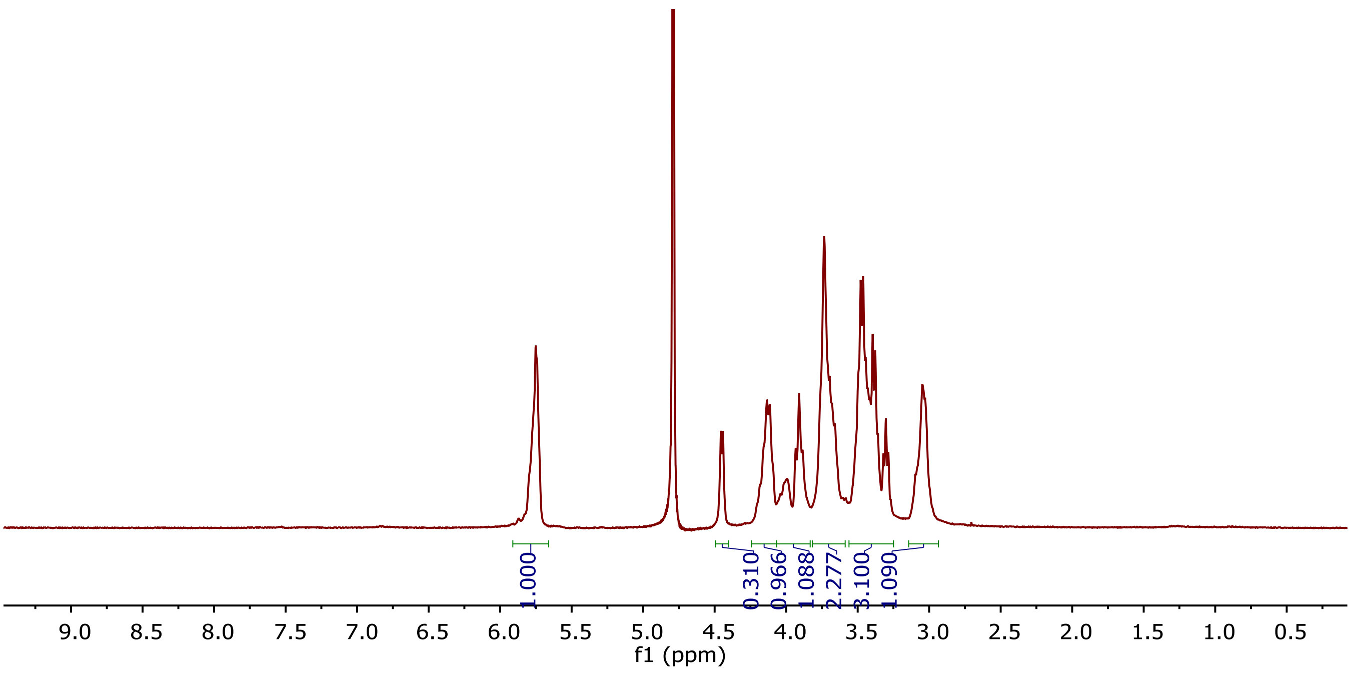

$^{1}$H NMR spectrum of P14'

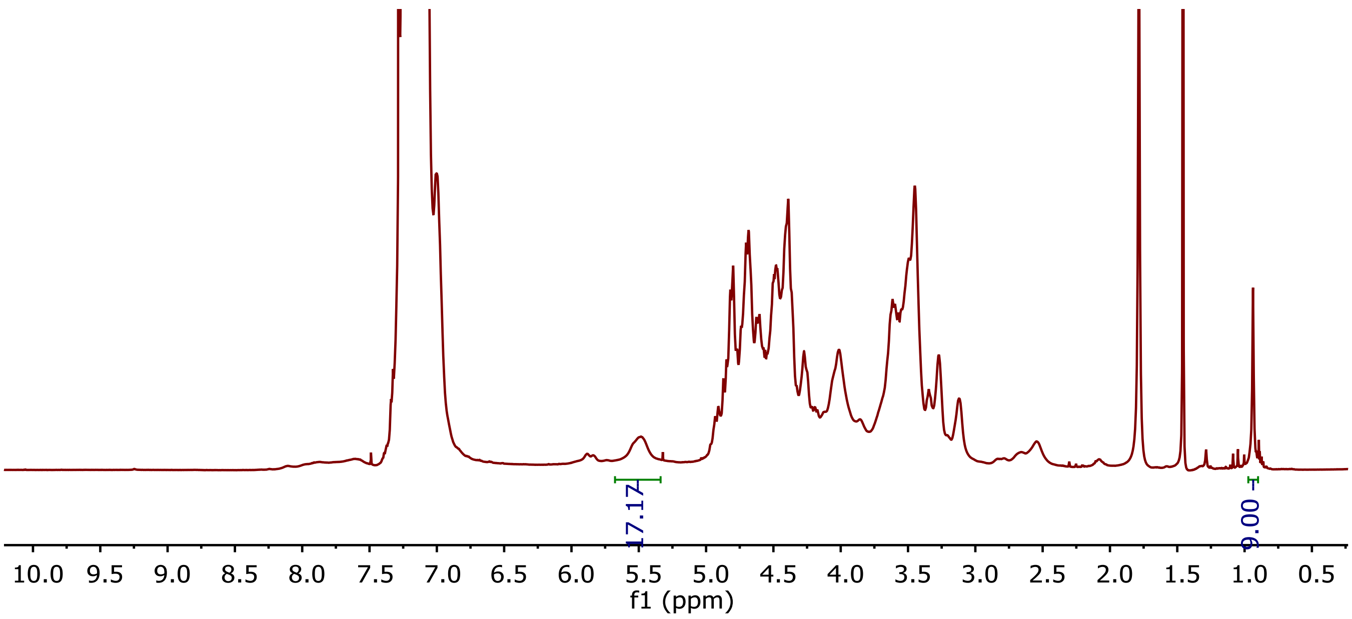


$^{1}$H NMR spectrum of P15'

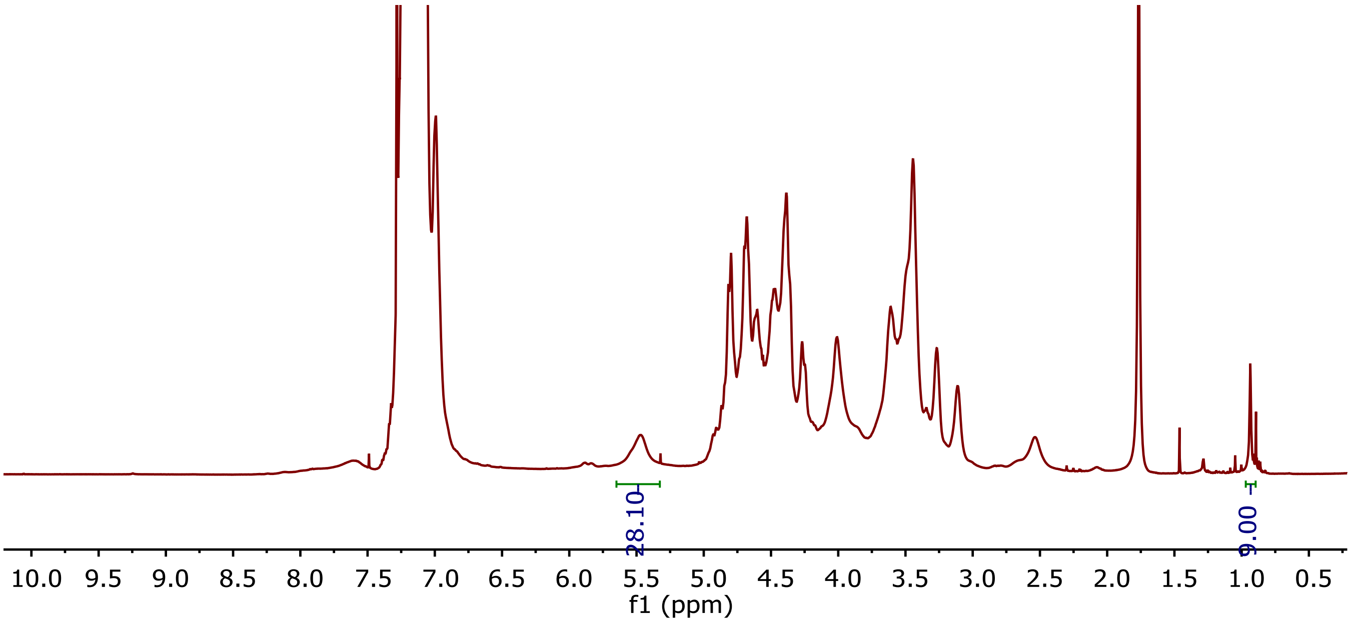

$^{1}H$ NMR spectrum of P16'

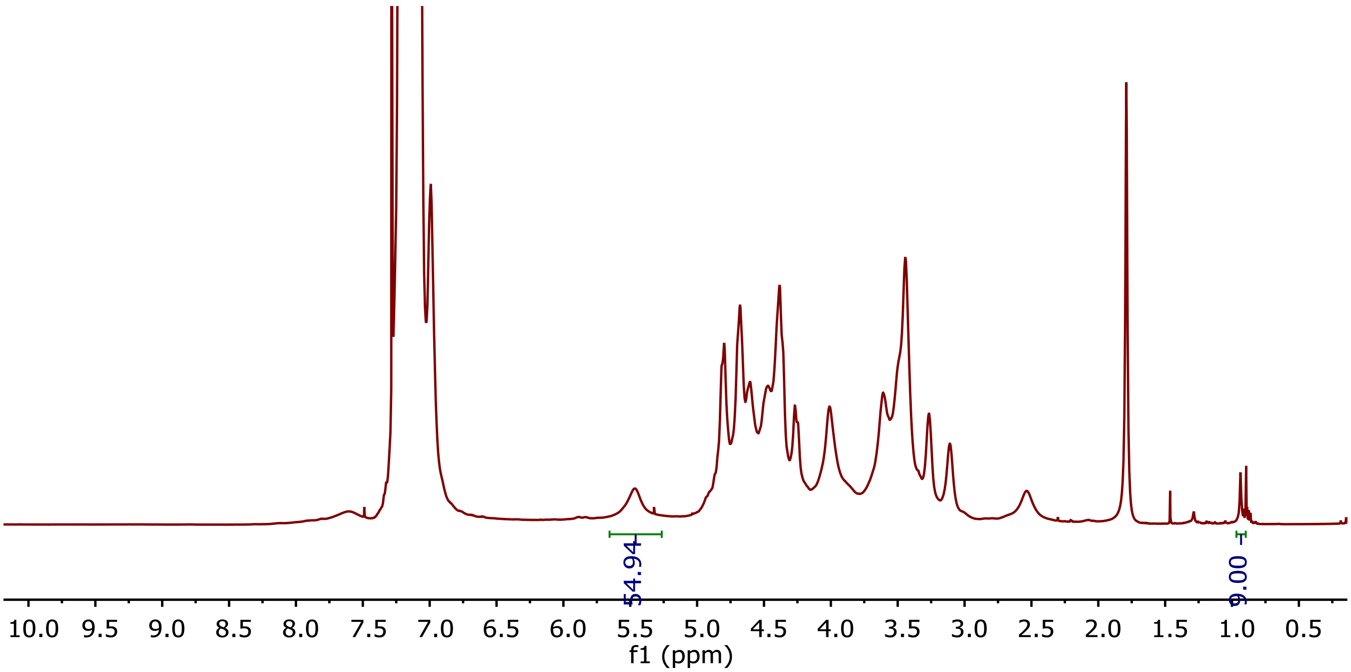


$^{1}H$ NMR spectrum of P14

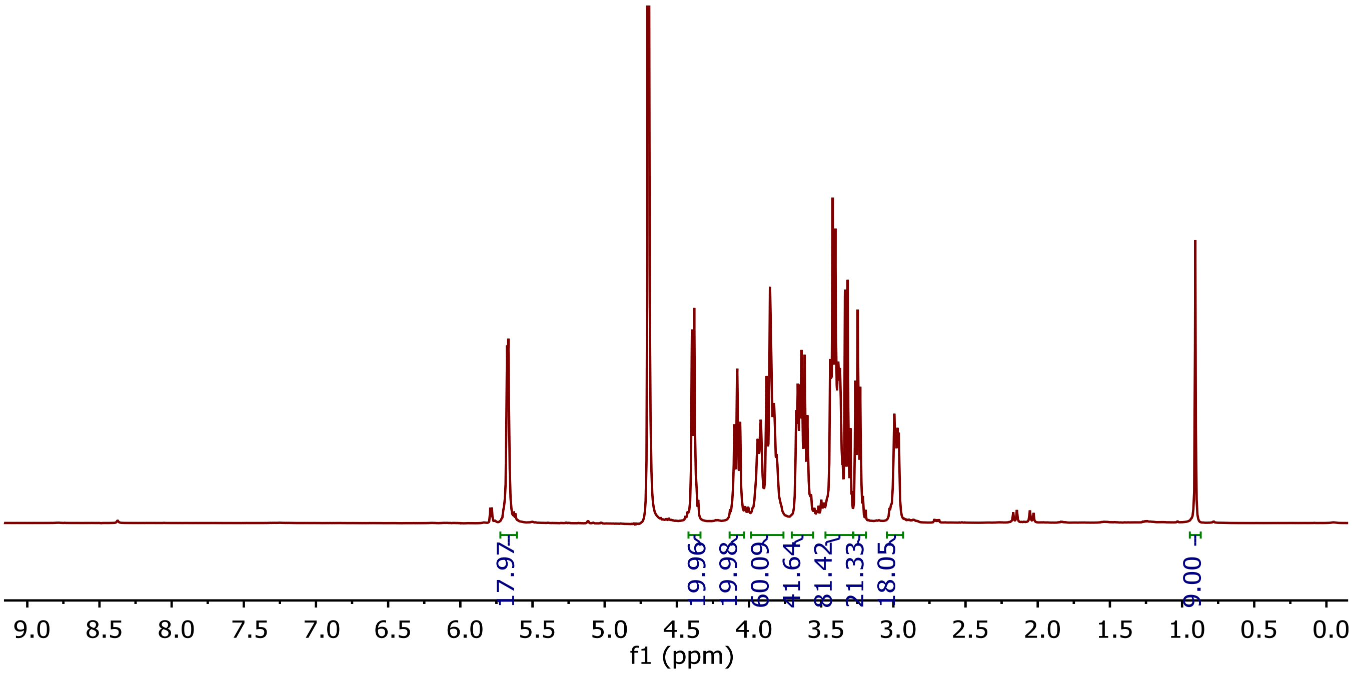

$^1$H NMR spectrum of P15

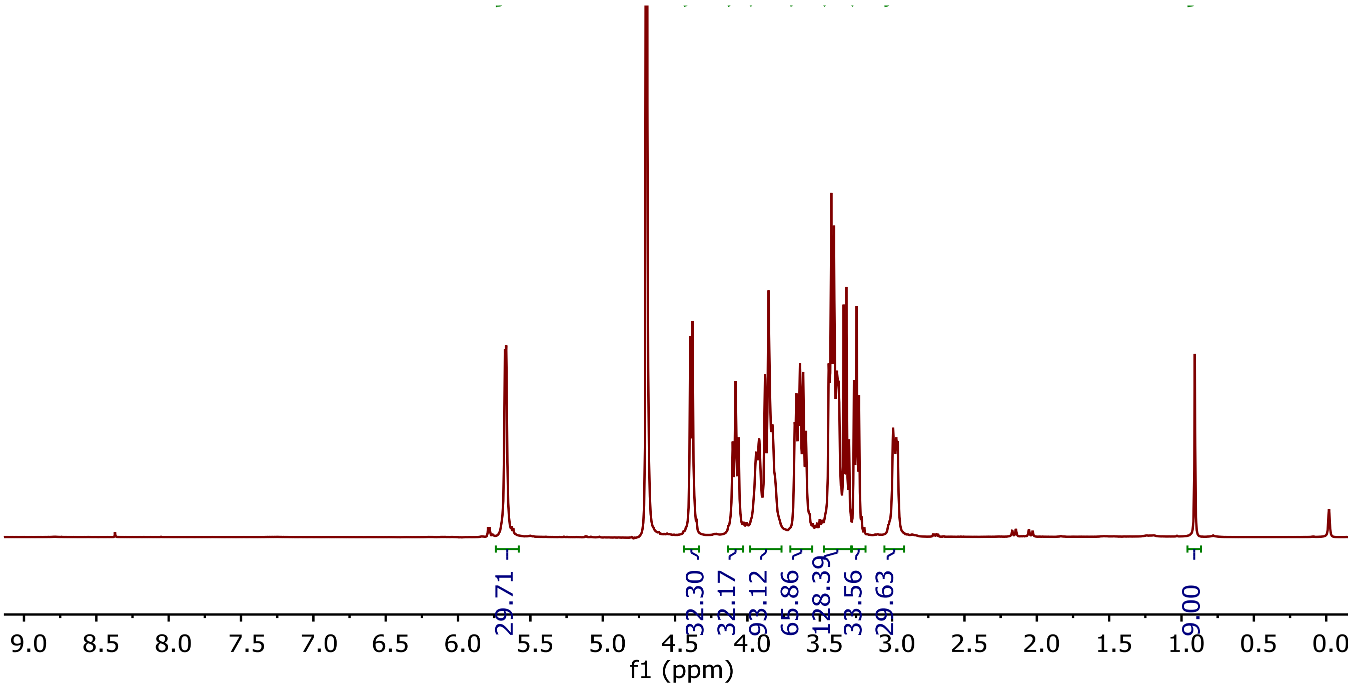


$^1$H NMR spectrum of P16

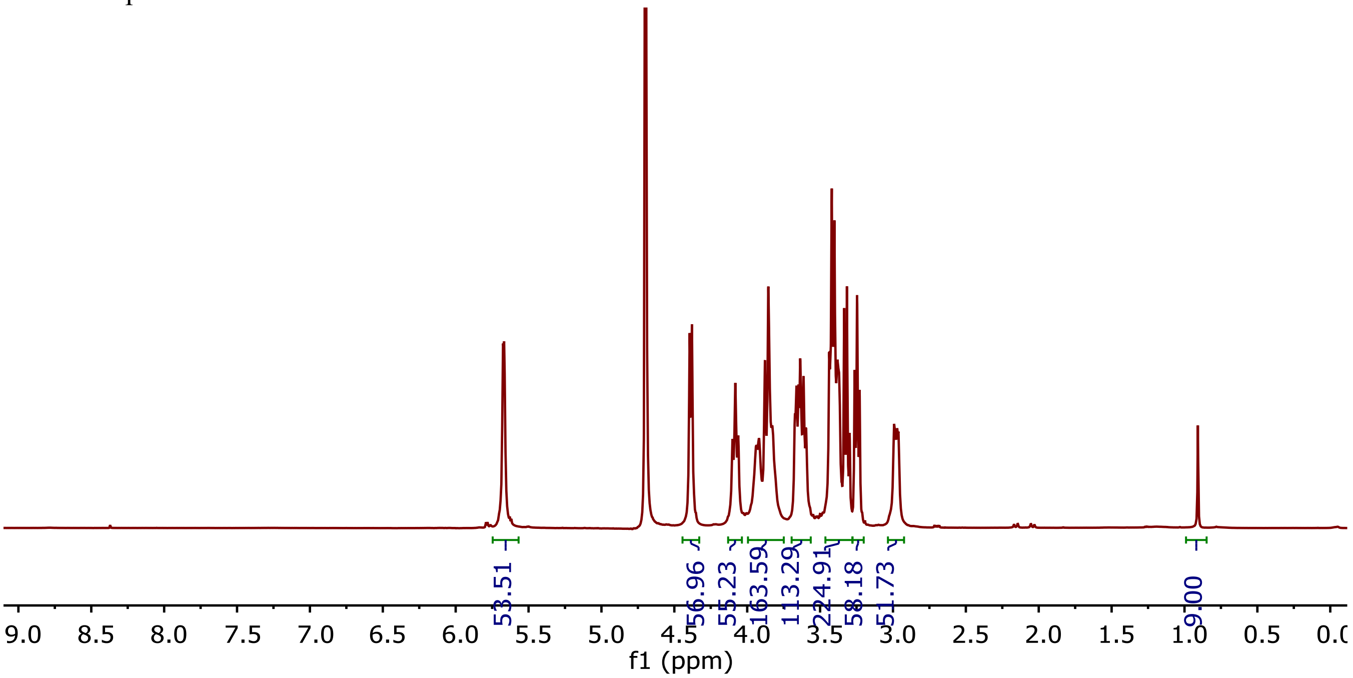

## Supplementary References


1. Brauns DH. Optical rotation and atomic dimension. VII. The halogeno-hepta-acetyl derivatives of gentiobiose. *J Am Chem Soc* **49**, 3170-3177 (1927).
2. Lee DJ, Harris PWR, Kowalczyk R, Dunbar PR, Brimble MA. Microwave-assisted synthesis of fluorescein-labelled GalNAc alpha 1-O-Ser/Thr (Tn) glycopeptides as immunological probes. *Synthesis*, 763-769 (2010).
3. Chmielewski M, Kaluza Z. [2 + 2] Cycloaddition of trichloroacetyl isocyanate to glycals. *Carbohydr Res* **167**, 143-152 (1987).
4. Satoh T, Kakuchi T. Synthesis of hyperbranched carbohydrate polymers by ring-opening multibranching polymerization of anhydro sugar. *Macromol Biosci* **7**, 999-1009 (2007).
