# Supplementary material for: Synthesis of bioactive (1→6)-β-glucose branched poly-amido-saccharides that stimulate and induce M1 polarization in macrophages
Source: Nat Commun. 2022 Aug 9;13:4661. doi: 10.1038/s41467-022-32346-5 (PMC9363418; doi:10.1038/s41467-022-32346-5)
Supplement: Supplementary file 3 — Reporting Summary [file 41467_2022_32346_MOESM3_ESM.pdf]

nature research

Corresponding author(s): Mark Grinstaff

Last updated by author(s): May 3, 2022

Reporting Summary

Nature Research wishes to improve the reproducibility of the work that we publish. This form provides structure for consistency and transparency in reporting. For further information on Nature Research policies, see our Editorial Policies and the Editorial Policy Checklist.

Statistics

For all statistical analyses, confirm that the following items are present in the figure legend, table legend, main text, or Methods section.

n/a	Confirmed	
☐	☒	The exact sample size (*n*) for each experimental group/condition, given as a discrete number and unit of measurement
☐	☒	A statement on whether measurements were taken from distinct samples or whether the same sample was measured repeatedly
☐	☒	The statistical test(s) used AND whether they are one- or two-sided<br>*Only common tests should be described solely by name; describe more complex techniques in the Methods section.*
☒	☐	A description of all covariates tested
☒	☐	A description of any assumptions or corrections, such as tests of normality and adjustment for multiple comparisons
☒	☒	A full description of the statistical parameters including central tendency (e.g. means) or other basic estimates (e.g. regression coefficient) AND variation (e.g. standard deviation) or associated estimates of uncertainty (e.g. confidence intervals)
☒	☒	For null hypothesis testing, the test statistic (e.g. *F*, *t*, *r*) with confidence intervals, effect sizes, degrees of freedom and *P* value noted<br>*Give P values as exact values whenever suitable.*
☒	☐	For Bayesian analysis, information on the choice of priors and Markov chain Monte Carlo settings
☒	☐	For hierarchical and complex designs, identification of the appropriate level for tests and full reporting of outcomes
☒	☐	Estimates of effect sizes (e.g. Cohen's *d*, Pearson's *r*), indicating how they were calculated

Our web collection on statistics for biologists contains articles on many of the points above.

Software and code

Policy information about availability of computer code

Data collection	Varian INOVA 500MHz spectrometer, Nicolet FTIR with a horizontal attenuated total reflectance (ATR) adapter plate, Tosoh EcoSEC Elite Model HLC-8420, Applied Photophysics CS/2Chirascan, and Rudolph Autopol II polarimeter
Data analysis	Biotek Synergy HT plate reader, Microsoft Excel, and Origin. All statistic analyses were performed in GraphPad Prism v9.

For manuscripts utilizing custom algorithms or software that are central to the research but not yet described in published literature, software must be made available to editors and reviewers. We strongly encourage code deposition in a community repository (e.g. GitHub). See the Nature Research guidelines for submitting code & software for further information.

Data

Policy information about availability of data

All manuscripts must include a data availability statement. This statement should provide the following information, where applicable:

- Accession codes, unique identifiers, or web links for publicly available datasets
- A list of figures that have associated raw data
- A description of any restrictions on data availability

The authors declare that the data supporting the findings of this study are available within the paper and its Supplementary information file. All other information is available from the corresponding authors upon reasonable request. Correspondence and requests for materials should be addressed to M.W.G.

Field-specific reporting

Life sciences study design

All studies must disclose on these points even when the disclosure is negative.

Sample size	The sample size was determined experimentally base on the variability of the results: n=3 for TNFa secretion, NO secretion NF-κB/AP-1, effect of antibody on the activation cell and cytotoxicity. Sample size was chosen based on literature and experience.
Data exclusions	No data was excluded from the data analysis.
Replication	All attempts at replication were successful. Replicates were performed independently and experimental findings were reproducible. Experiments were performed in triplicate.
Randomization	The nature of the experiments in our work do not require randomization, due to the specific in vitro assays performed.
Blinding	Blinding was no performed for the in vitro cell assays.

Reporting for specific materials, systems and methods

We require information from authors about some types of materials, experimental systems and methods used in many studies. Here, indicate whether each material, system or method listed is relevant to your study. If you are not sure if a list item applies to your research, read the appropriate section before selecting a response.

Materials & experimental systems

n/a	Involved in the study	
☐	☒	Antibodies
☐	☒	Eukaryotic cell lines
☒	☐	Palaeontology and archaeology
☒	☐	Animals and other organisms
☒	☒	Human research participants
☒	☐	Clinical data
☒	☐	Dual use research of concern

Methods

n/a	Involved in the study	
☒	☐	ChIP-seq
☒	☐	Flow cytometry
☒	☐	MRI-based neuroimaging

Antibodies

Antibodies used	Antibodies used in this study were Anti-mDectin-1-IgG antibody and Rat IgG2a (InvivoGen), mabg-mdect .
Validation	We only used commercially avalaible antibodies. No validation was done in our laboratory beyond the use of a negative control.

Eukaryotic cell lines

Policy information about cell lines

Cell line source(s)	RAW264.7, RAW-Blue, Sarcoma-180, THP-1, L929, and HepG2 cells were obtained from ATCC. We cultured peripheral blood mononuclear cells (PBMCs) isolated from patient blood samples obtained at Boston Children's Hospital's Plasma Donation Center.
Authentication	We did not perform authentication of the cell lines.
Mycoplasma contamination	We performed mycoplasma contamination analysis, and the cells were free of mycoplasma.
Commonly misidentified lines (See ICLAC register)	No commonly misidentified lines were used in this study.
